# Whole genome bisulfite sequencing methylome analysis of mulberry (*Morus alba*) reveals epigenome modifications in response to drought stress

**DOI:** 10.1038/s41598-020-64975-5

**Published:** 2020-05-15

**Authors:** Ruixue Li, Fei Hu, Bing Li, Yuping Zhang, Ming Chen, Tao Fan, Taichu Wang

**Affiliations:** 1 Sericultural Research Institute, Anhui Academy of Agricultural Sciences, Hefei, Anhui 230061 China; 2 Plant Protection and Agroproducts Safety Institute, Anhui Academy of Agricultural Sciences, Hefei, Anhui 230031 China

**Keywords:** Epigenetics, Plant molecular biology, Plant stress responses

## Abstract

DNA methylation plays a significant role in many biological processes. Although some studies of DNA methylation have been performed in woody plant, none is known about the methylation patterns of mulberry (*Morus alba*). In this study, we performed whole genome bisulfite sequencing under drought stress to generate a methylated cytosines map and assessed the effects of the changes on gene expression combined with transcriptomics. We found that the percentage of methylated cytosines varied depending on the local sequence context (CG, CHG and CHH) and external treatment (control, CK; drought stress, DS). The methylation levels under DS were 8.64% higher than that of CK, and differences that were mainly due to the contribution of mCG (6.24%). Additionally, there were 3,243 different methylation and expression associated genes. In addition, methylated genes were enriched within GO subcategories including catalytic activity, cellular process, metabolic process, response to stimulus and regulation of biological process. This is the first study to comprehensively present methylation patterns in mulberry and reveal widespread DNA methylation changes in response to drought stress, which has the potential to enhance our understanding of links between DNA methylation and the modulation of gene expression in plants subjected to abiotic stresses.

## Introduction

Epigenetics is the study of heritable changes in gene expression that occur independently of DNA sequence changes^1–3^. As one of the defense mechanisms used by plants, epigenetics facilitates the genomic “plasticity” of plants and plays significant roles in growth, development and adaption to stress^4–7^. DNA methylation is a common feature of eukaryotic genomes and is one of the main types of epigenetic modifications. It plays a significant role in regulation of gene expression, genomic imprinting and transposon silencing, which has a protective effect on genomic integrity^8–10^. In plants, the DNA methylation patterns of different species are diverse. In general, methylation occurs at CG, CHG, and CHH (H = A, C, or T) nucleotides of genes and transposable elements (TEs) in angiosperms^11^. In each of these contexts, distinct mechanisms are involved in establishing, maintaining and removing methyl groups. In the model plant *Arabidopsis*, which has a compact genome and rapid generation time, the first whole-genome methylome map was deciphered^12,13^. Now, many reports have documented the importance of DNA methylation in the process of plant growth and development, defense, and response abiotic stress, which includes the response to salt, drought and heavy metal exposure^14–16^. In long-term subcultures, plant growth regulator (PGR)-supplementation gradually increased global DNA methylation in *Araucaria angustifolia*, which compromises genomic stability and alters gene expression^17^. Induction of the *OsMYB91* gene in rice in response to salt stress was associated with the rapid removal of DNA methylation from the promoter region of the gene and histone modification of the locus, which suggested that dynamic methylation patterns may have a role in modulating the expression of the gene^18^.

Drought is a major type natural disaster. With the intensification of the greenhouse effect, dry weather is predicted to occur increasingly frequently. This will likely result in huge losses with respect to industrial and agricultural production, and serious damage to the ecological environment. The plant response to drought stress is a complicated process, which involves various physiological, biochemical, and genetic responses. Among these, gene expression, metabolic networks, and DNA methylation are particularly important. Researchers have shown that drought induces changes to DNA methylation patterns in variety specific, tissue specific and stress specific ways^19,20^. The amplitude of drought-related transcriptional change was related to the extent to which genomic changes in DNA methylation of poplar (*Populus trichocarpa*) occurred, suggesting the importance of epigenetic mechanisms in the adaption of long-lived trees to local environments^21^. When comparing two varieties of fava bean (*Vicia faba*), researchers determined that DNA methylation of root tissues decreased 3.63% (from 23.43% to 19.80%) under drought stress in drought-tolerant Bachar and increased 0.66% (from 16.53% to 17.19%) in the drought-sensitive F177 variety^22^. Drought stress strongly induced de novo methylation (DNM) within the genome of sesame (*Sesamum indicum*), and most of the methylated loci were demethylated (DM) during the recovery phase^23^. Analysis of methylation patterns in two contrasting wheat genotypes revealed that demethylation occurred more frequently in the drought-tolerant genotype (C306) than the drought-sensitive genotype (HUW468) post-exposure to drought stress. Comparisons of different methylation-sensitive amplified polymorphism (MSAP) patterns showed that there was a percentage of polymorphic bands which occurred between tolerant and susceptible wheat genotypes (from 74.79% at anthesis to 88.89% at tillering)^24^. These studies revealed a direct relationship between environmental drought stress and DNA methylation changes.

Mulberry (*Morus alba*) is an important perennial economic tree that has a broad ecological distribution in China. Aside from its use in sericulture, mulberry has important economic and ecological value. Mulberry has a high degree of adaptability to poor environmental conditions including drought, cold, high salt, waterlogging, and heavy metal ion exposure^25,26^. Currently, the genome and the transcriptome of mulberry tree under drought stress have been determined^27,28^. The availability of high-quality reference genomes and improvements in sequencing technologies have facilitated whole-genome methylome analyses of mulberry and other plant genomes, such as the recently discovered rice DNA methylation pathways^29^, cytosine methylation that occurs in apple, which is associated with water deficit^30^, and single-base resolution methylomes of upland cotton (*Gossypium hirsutum*)^31^. However, there have been no studies that have assessed of mulberry methylome and the influence of adversity stress, such as drought stress, on mlberry methylome. Here, we generated a genome-wide, high-coverage DNA methylation maps using whole genome bisulfite sequencing (WGBS)^32,33^ in mulberry under normal and drought stressed conditions to investigate whether whole genome epigenome reprogramming occurs as a result of drought stress in mulberry. This study aimed to: (1) determine the genome-wide scale mulberry methylome level; (2) appraise changes of mulberry methylome associated with drought stress; (3) evaluate of the relationship between changes in the methylome and drought-stress associated changes in gene expression. This study has the potential to greatly enhance our understanding of the impact of level differentially methylated regions on gene expression and drought stress response, and provides a means to explore the basis of resistance to facilitate mulberry breeding.

## Results

### Bisulfite sequencing summary and QC

In order to identify the overall methylation patterns of mulberry plants in response to drought stress at a global level, six mulberry samples (3 control samples (CK) and 3 drought stressed samples (DS)) were sequenced using WGBS technology using Illumina Hi-Seq. 2500 platforms (BGI, Shenzhen, China). Then, the corresponding methylomes were decoded and analysed. Our data have been deposited in the NCBI Sequence Read Archive (SRA) (https://ncbi.nlm.nih.gov/subs/sra) with an accession number of PRJNA579451. Up to ~70 million sequencing reads were generated for each replicate and ~12.15 Gb clean bases representing >20-fold coverage of the NCBI reference genome were generated after filtering low quality reads, N reads and adaptor sequences. The clean rates from six mulberry samples were 87.29% to 89.96% (Table S1). After filtering, clean reads were mapped to the reference sequence using BSMAP software^34^. The genomic mapping rate and descriptions of genomic coverage were included in Table 1. The mapping rate was 42.51%, indicating that the method provided results with high reliability and accuracy. The effective data coverage of C sites provided information regarding the methylation states of individual cytosines with high confidence. The percentage of methylated cytosines varied depending on the local sequence context (C, CG, CHG and CHH) and the external treatment provided (CK and drought stress). Moreover, the effective coverage of the whole genome was as much as ~46.77% and there were obvious difference between regulatory element types, as shown in Table 1 and Table 2. In addition, the distribution of methylation sites within each chromosome was analysed (Table S2).

**Table 1:** Alignment statistics with reference genome.

Sample ID	Clean Reads	Mapped Reads	Mapping Rate (%)	Bisulfite Conversion Rate (%)	Duplication Rate (%)	Average Depth (X)	Coverage (%)
CK1	69,846,568	29,737,351	42.58	99.56	13.04	9.83	47.116
CK2	90,293,876	39,060,708	43.26	99.57	14.05	12.71	48.249
CK3	82,396,506	35,310,978	42.85	99.53	14.34	11.51	47.931
DS1	90,936,240	40,008,819	44.00	99.58	15.68	12.45	47.620
DS2	73,026,990	31,043,459	42.51	99.58	14.95	9.98	47.727
DS3	79,672,832	34,528,597	43.34	99.56	19.05	10.42	46.771

**Table 2:** Effective coverage of whole genome and some types of elements.

Sample	Region	C (%)	CG (%)	CHG (%)	CHH (%)
CK	Whole Genome	43.89	41.35	46.38	44.18
	CDS	84.15	82.57	85.31	84.27
	Down2k	43.80	41.63	46.29	43.73
	Up2k	39.20	38.12	41.46	39.02
	exon	84.15	82.57	85.31	84.27
	mRNA	71.46	70.64	75.21	70.81
	repeat	45.35	43.72	44.94	45.68
	CpGIsland	41.77	43.07	41.46	41.17
DS	Whole Genome	43.17	41.00	45.89	43.06
	CDS	83.76	82.56	85.11	83.73
	Down2k	42.96	41.34	45.86	42.74
	Up2k	38.25	37.69	40.92	37.91
	exon	83.76	82.56	85.11	83.73
	mRNA	70.76	70.46	74.80	69.94
	repeat	45.14	44.03	45.22	45.31
	CpGIsland	42.35	43.77	42.15	41.64

Note: CDS: Coding sequence; Down2k: 2 kb downstream regions from genes; Up2k: 2 kb upstream regions from genes; repeat: genome repeat sequence.

### Genome-wide patterns of DNA methylation in mulberry

**Figure 1:**
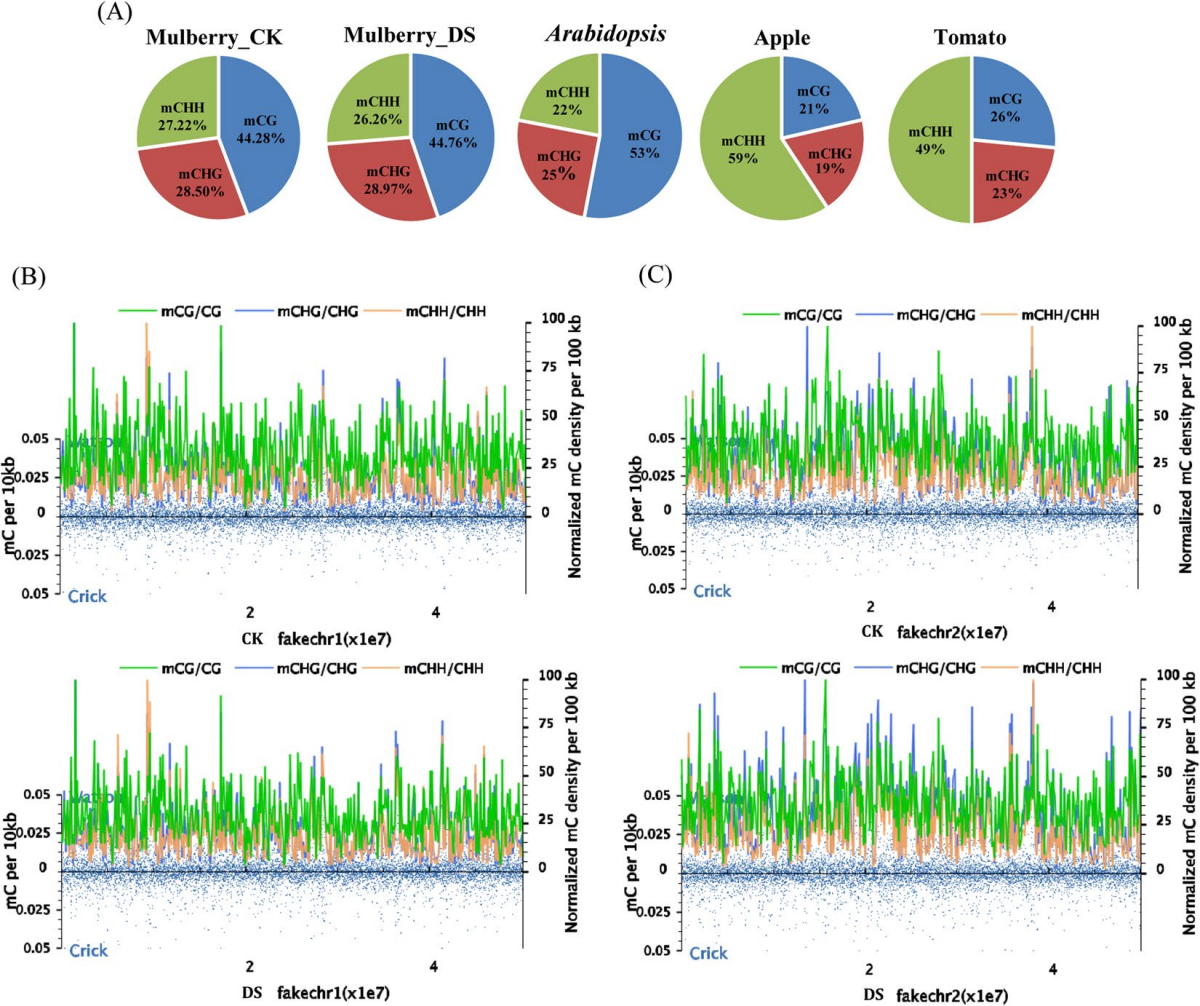
The mulberry epigenome. (**A**) Relative proportion of cytosine methylation in three different sequence contexts (mCG, mCHG and mCHH) in mulberry, apple, *Arabidopsis* and tomato^31^. (**B-C**) Distribution of cytosine methylation throughout chromosome 1 and 2. Blue dots indicate methyl-cytosine density within 10-kb windows distributed throughout chromosome 1 (the black rectangle of the horizontal axis represents the centromere). Smooth lines represent the methyl-cytosine density for each context.

The average methylation levels of the whole genome revealed the general characteristics of methylome and was determined by the ratio of the number of reads supporting methylation to the number of reads covering certain cytosine site. The percentage of methylated cytosines varied depending on the local sequence context (C, CG, CHG and CHH) and the external treatment provided (CK and drought). The CK-treated genome had methylation levels of ~9.95% (mCs), 33.03% (mCG), 20.63% (mCHG) and 2.86% (mCHH) for total sequenced C, CG, CHG and CHH sites, respectively, which reflected the percentage of methylation levels in the genome. Accordingly, DS-treated plants had methylation of ~10.81%, 35.09%, 20.96% and 2.98% in C, CG, CHG and CHH contexts (Table 3 and Table S3) showing that methylation levels were generally up-regulated after drought stress compared with controls. We also analysed the proportions of mCG, mCHG and mCHH sequences relative to the occurrence of total mC sites, which reflected the distributions of mCs in the three sequence contexts. As shown in Fig. 1A, methylcytosine was most often found at mCG sites (44.28%), and occurred less frequently at mCHG and mCHH sequences (28.50% and 27.22%, respectively). Drought stress was associated with a slight increases in the proportions of mCG and mCHG methylation, and a decrease in mCHH methylation in mulberry (Fig. 1A and Table S4). These data showed that drought stress induced methylation in two sequence contexts, including mCpG and mCHG, and displayed hyper-methylation pattern after drought stress.

**Table 3:** Average methylation level of whole genome and every chromosome.

Sample	Chromosome	mC (%)	mCG (%)	mCHG (%)	mCHH (%)
CK	Whole Genome	9.95 ± 0.21	33.03 ± 0.33	20.63 ± 0.24	2.86 ± 0.13
	fakechr1	8.70 ± 0.22	28.74 ± 0.46	17.43 ± 0.29	2.50 ± 0.11
	fakechr2	10.72 ± 0.23	35.99 ± 0.35	23.10 ± 0.25	3.08 ± 0.14
	fakechr3	10.30 ± 0.21	35.09 ± 0.31	20.96 ± 0.23	2.98 ± 0.14
	fakechr4	9.34 ± 0.20	31.32 ± 0.31	19.42 ± 0.21	2.75 ± 0.12
	fakechr5	11.01 ± 0.22	34.59 ± 0.34	23.21 ± 0.25	3.05 ± 0.15
	fakechr6	9.96 ± 0.19	33.84 ± 0.21	20.58 ± 0.19	2.92 ± 0.13
	fakechr7	9.19 ± 0.19	31.16 ± 0.21	18.50 ± 0.22	2.57 ± 0.12
DS	Whole Genome	10.81 ± 0.26*	33.82 ± 0.65	21.95 ± 0.32**	3.25 ± 0.29
	fakechr1	9.35 ± 0.23*	29.11 ± 0.83	18.44 ± 0.37**	2.81 ± 0.24
	fakechr2	11.67 ± 0.29*	36.85 ± 0.69	24.69 ± 0.36**	3.51 ± 0.32
	fakechr3	11.28 ± 0.28*	36.32 ± 0.57*	22.49 ± 0.33**	3.40 ± 0.30
	fakechr4	10.30 ± 0.24*	32.45 ± 0.45*	21.07 ± 0.26**	3.18 ± 0.28
	fakechr5	11.73 ± 0.29*	34.94 ± 0.99	23.79 ± 0.43	3.43 ± 0.32
	fakechr6	10.91 ± 0.26*	34.72 ± 0.34*	22.32 ± 0.21**	3.34 ± 0.30
	fakechr7	10.02 ± 0.26	32.01 ± 0.01	19.87 ± 0.18**	2.92 ± 0.27

Note: The asterisk behind the results indicate the significant difference of the changes between CK and DS samples with the level of *P* > 0.05 (shown as nothing), 0.01 < *P* ≤ 0.05 (shown as *) and *P* ≤ 0.01 (shown as **). The same as below.

As shown in Fig. 1B-1C and Fig. S1-S5, a global-scale view of DNA methylation levels revealed that the density of DNA methylation levels of each chromosome varied widely. The cytosine methylation level of mC, mCG, mCHG and mCHH sequences throughout chromosome 1 and 7 were all down-regulated under drought stress compared with the control, and mC methylation level throughout chromosome 4 and 6 were up-regulated. The mCHG methylation level throughout chromosome 2 showed up-regulated pattern and mCG methylation level throughout chromosome 3 showed down-regulated pattern, and there were no obvious changes on other mC sequences and other chromosome. Moreover, sub-telomeric regions of the chromosomes had greater DNA methylation densities, which might be important for the control of telomere length and recombination. Fig. S6 showed percentages of methyl-cytosine according to methylation levels. In the mulberry genome, most of the mCG methylation sites were 80%-100% methylated, which corresponds to high levels of methylation. mCHG sites were ranged from 20% to 100% methylated, and displayed a relatively flat trend. The methylation levels of mCHH sites were relatively low and were generally within the 20% to 30% range. The percentage of mC methylation level with below 80% in DS was higher than that in CK (0.01 < *P* ≤ 0.05). There was no obvious difference of the percentage of mCG and mCHG methylation level between CK and DS (*P* > 0.05). From Table 3 we could see that the methylation level in whole genome and seven chromosomes all exhibited an up-regulated pattern under drought stress compared with the control. Interestingly, we found that the mCHH methylation in whole genome and seven chromosomes showed highest up-regulated pattern with 12.40–15.64% range among mC, mCG, mCHG and mCHH sequences, but the mCHH methylation level of CK and DS in whole genome and seven chromosomes had no obvious difference (*P* > 0.05) and the mCHG methylation level of CK and DS had significant difference (*P* ≤ 0.01) except for chromosomes fakechr5. This suggested that the mulberry methylation level was dynamic with environments and that mCHG methylation may be very closely correlated with drought stress.

### DNA methylation patterns in different regions of the mulberry genome

To explore the DNA methylation patterns in different regions of the mulberry genome, we analysed the methylation profiles within genes, including coding sequences and introns. In each context, and in both CK and DS genomes, methylation levels of mCG were highest and levels of mCHH methylation were lowest when the three CG, CHG and CHH types of methylation sites were compared. This was true for different genomic regions of mulberry (Table 4). mCG methylation was highest in transcriptional termination regions (TTRs, 2 kb downstream, Down2k) regions from genes and lowest in the coding sequences (CDS) and exons. mCHG and mCHH methylation was highest within CpG islands and lowest in the CDSs and exons. On the whole, there was no significant difference (*P* > 0.05) in different regions of the mulberry genome between CK and DS except for mC methylation in CpGIsland (*P* ≤ 0.01), mCG methylation in CpGIsland (0.01 < *P* ≤ 0.05) and mCHG methylation in Up2k (0.01 < *P* ≤ 0.05).

**Table 4:** Average methylation levels of some types of elements.

Sample	Region	mC (%)	mCG (%)	mCHG (%)	mCHH (%)
CK	CDS	3.57 ± 0.03	14.50 ± 0.06	2.55 ± 0.03	0.78 ± 0.01
	Down2k	5.70 ± 0.22	18.19 ± 0.62	11.06 ± 0.35	1.81 ± 0.07
	Up2k	5.72 ± 0.21	16.89 ± 0.54	11.63 ± 0.33	1.77 ± 0.07
	exon	3.57 ± 0.03	14.50 ± 0.06	2.55 ± 0.02	0.78 ± 0.01
	CpGIsland	8.82 ± 0.18	17.44 ± 0.29	12.60 ± 0.27	2.11 ± 0.09
DS	CDS	3.65 ± 0.08	14.27 ± 0.37	2.61 ± 0.14	0.80 ± 0.02
	Down2k	5.94 ± 0.35	18.70 ± 0.49	10.85 ± 0.65	1.89 ± 0.12
	Up2k	5.83 ± 0.45	16.89 ± 0.69	11.80 ± 0.33*	1.82 ± 0.11
	exon	3.65 ± 0.08	14.27 ± 0.37	2.61 ± 0.14	0.80 ± 0.02
	CpGIsland	9.37 ± 0.19**	18.69 ± 0.53*	12.58 ± 0.24	2.32 ± 0.17

**Figure 2:**
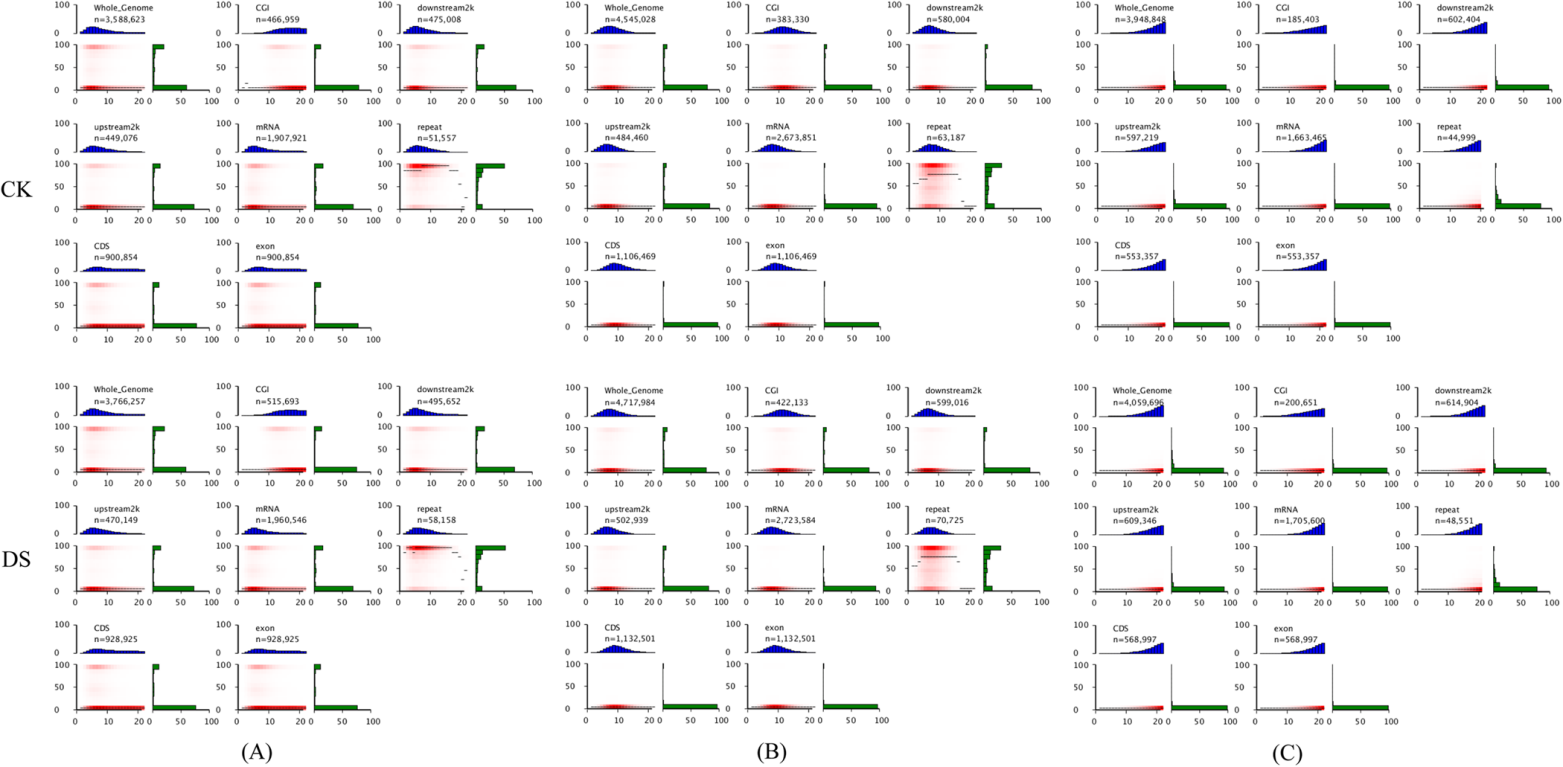
Heat maps show distinct methylation and CpG density patterns. CpG density (x-axis) is defined as number of CpG dinucleotides occurring within 200 bp windows. Methylation level (y-axis) is defined as the average cytosine methylation level of CpG sites. Thin black lines within each heat map denote the median methylation level of CpGs at the given local density. The red colour gradient indicates abundance of CpGs that fall into bins of given methylation levels and CpG densities. Blue bar charts above each heat map show the distribution of CpG densities, which are projected onto the x-axis of the heat maps, n represents the number of 200 bp windows that used to calculate. The green bar charts to the right of the heat maps show the distribution of methylation levels, which are projected onto the y-axis of the heat maps. (**A**), (**B**) and (**C**) represent CG, CHG and CHH sequences, respectively.

We further evaluated the proportion of mCG, mCHG and mCHH observed relative to total mC occurring within distinct genomic elements (Fig. 2), which can help to identify the function of methylation changes for each element in response to drought stress. The levels of mCG, mCHG and mCHH observed varied among genomic elements. mCG methylation accounted for approximately half of the total mC observed, and mCHH occurred least frequently in each genomic region and under both treatment (CK and DS), which suggested that mCG sites are the most important type among the three sequence contexts examined. mCG methylation within the promoters 2 kb upstream (Up2k) of genes and Down2k (TTR) regions was much higher than within CDS and exons. Further, methylation levels in exon and CDS were much lower than CpG islands. Genome repeat sequences were the most highly methylated among genomic regions assessed. The results suggested that cytosine methylation levels are correlated with gene element type. Methylation levels of mCHG and mCHH sites showed similar patterns as those observed for mCG sites. We then compared the methylation levels of different genomic elements between CK and DS, and found that methylation levels of different genomic elements in DS were higher than them in CK.

**Figure 3:**
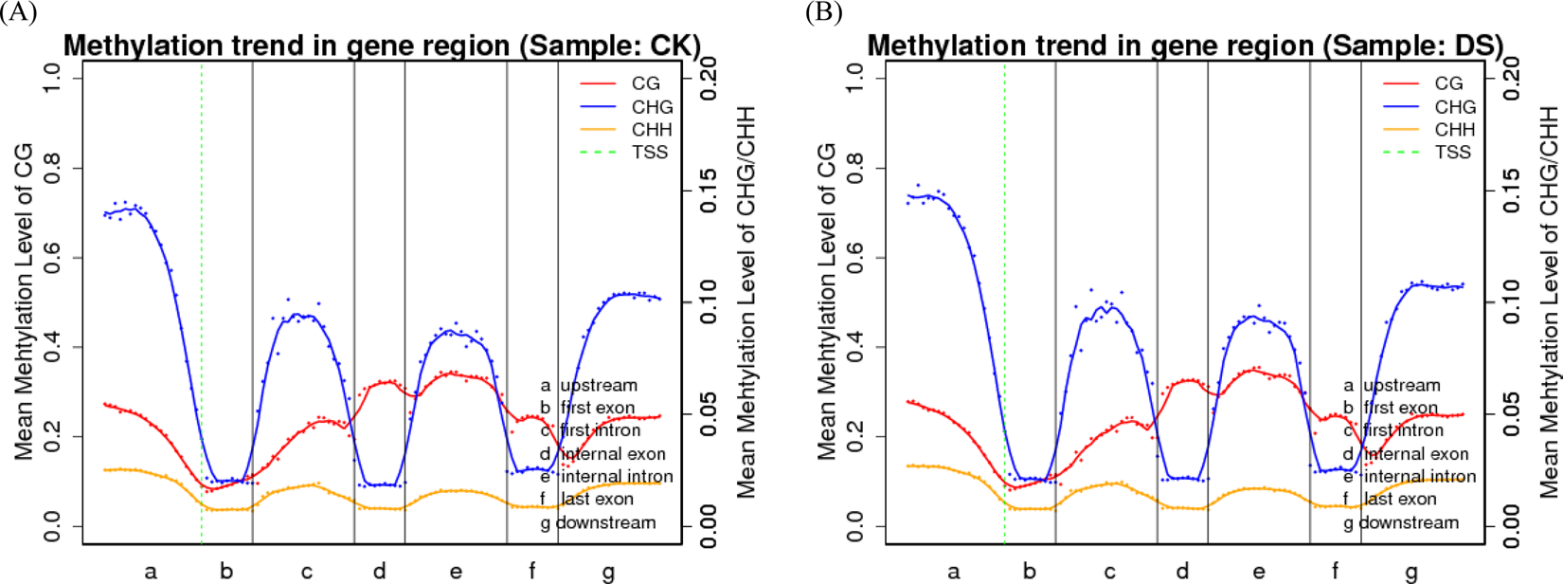
Canonical DNA methylation profiles of the entire transcriptional units. The canonical gene structure is defined by 7 different features, which are displayed on the x-axis. The length of features were normalised and partitioned into equal numbers of bins. Each dot denotes the mean methylation level per bin and lines denote 5-bin moving averages. The green vertical lines indicate the mean locations of transcriptional start sites.

In order to reveal the relationship between DNA methylation profiles and genes expression in detail, canonical DNA methylation profiles of the entire transcriptional units were divided into 7 distinct functional elements to study the changes of methylation level. As shown in Fig. 3, among regularity of mCG, mCHG and mCHH methylation sites, the distribution of the mCG methylation sites in 7 distinct genomic functional elements showed strongest regularity. The mCG methylation sites showed high methylation enrichments in upstream, transcription initiation site (TSS) upstream, first intron, internal exon, internal intron, last exon and downstream elements with comparative low methylation status among TSS downstream and first exon. The mCHG and mCHH methylation sites had the some regularity and showed high methylation enrichments in upstream, TSS upstream, first intron, internal intron and downstream elements with comparative low methylation status among TSS downstream, first exon, internal exon and last exon elements. In addition, mCG methylation level of DS samples were elevated among the distinct functional elements compared to CK samples except for first intron.

### Cytosine DNA methylation may have sequence preference

**Figure 4:**
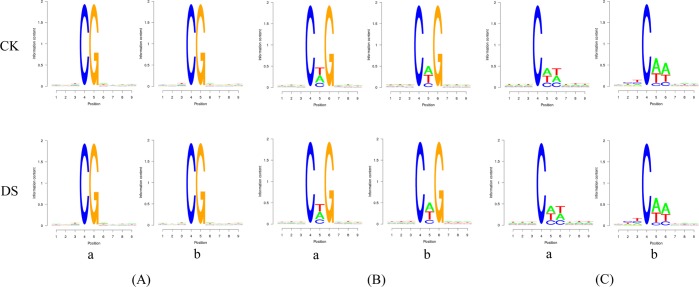
Sequence preference for cytosine DNA methylation. Weblogo analysis of bases surrounding mC sites within different sequence contexts. The X axis represents the position of bases in which the methylated cytosine is placed in the fourth position. The Y axis indicates the entropy of the base. (**A**), (**B**) and (**C**) represent CG, CHG and CHH sequence contexts, respectively. Further, a and b represent all C sites and mC sites, respectively.

In eukaryotes, the sequence features of bases near methylation site play a guiding significance on reflecting sequence preference of the methylation^13^. To reveal whether there was a relationship between sequence context and methylation preference in mulberry, we calculated the percentage methylation of all 9 bp sequences in which methylated cytosines were located in the fourth position using a tool named Logo Plots to explore sequence information for a methylated site or its nearby region. At the same time, the genome-wide cytosine methylation patterns were also analysed. The entropy of base was 0 as the minimum value, which indicated the four bases were evenly proportioned with 25%; the entropy of base was 2 as the maximum value, which indicated the four bases were most unevenly distributed and only a specific bases appeared, such as the fourth of C and the fifth of G. In the mulberry genome, the sequence preference around methylated sites were differed depending on mCG and non-CG context for all methylated mC sites (Fig. 4). In the mCG context, mC frequently occurred at TCGA sequences, while methylation of mC sites within the mCHG context occurred most frequently at CAG sequences. Methylation within the mCHH context occurred most frequently in CAA sequences. Therefore, we inferred that most methylation at mC sites occurred within the CAN (N represents A, T, C, G) context.

### Differentially methylated regions (DMRs) responding to drought stress

Differentially methylated regions (DMRs) are stretched of DNA in a sample’s genome that have different DNA methylation patterns compared with other samples. To reveal the potential role of methylation in response to drought stress, we analysed DMRs of drought-stressed mulberry plants compared with normally grown mulberry plants. A sliding-window approach was used to identify DMRs that contained at least five CG (CHH or CHG) sites and the methylation levels from the two different samples assessed differed^35^. In total, 18,580 DMRs were identified from mulberry plants subjected to drought stress compared with normal growing condition, and there were 15,088, 3,487 and 5 DMRs in mCG, mCHG, mCHH contexts, respectively. These included 9,027 hypermethylated DMRs and 9,553 hypomethylated DMRs after drought stress, which indicated that some DMRs were dynamically changed as a result of exposure to environmental stress. We also performed an analysis of DMR length for each chromosome (Table 5) and found that the length of DMRs varied among the 7 chromosomes of mulberry. When assessing mCG and mCHH patterns, the longest DMRs were found in fakechr1, while for mCHG patterns, the longest DMRs were found in fakechr3. These results may be correlated with numbers of genes, transposons and the occurrence of other elements within each chromosome. Above all, we inferred that plants can alter methylation levels or methylation patterns in certain genes and pathways related to stress. This helps plants to regulate gene expression in response to stress.

**Table 5:** Identification of DMRs and an analysis of CK_vs_DS.

Chromosome	mCG pattern		mCHG pattern		mCHH pattern	
	DMRs number	DMRs length	DMRs number	DMRs length	DMRs number	DMRs length
fakechr1	2479	1160830	493	184215	1	2167
fakechr2	2278	1061704	570	212437	0	103
fakechr3	2467	1145600	612	242591	0	158
fakechr4	2424	1120986	514	189430	1	231
fakechr5	2246	1034223	515	187318	0	74
fakechr6	2421	1114354	587	226308	1	483
fakechr7	771	357295	196	77377	0	68
Total	15088	6994992	3487	1319675	5	1333

**Figure 5:**
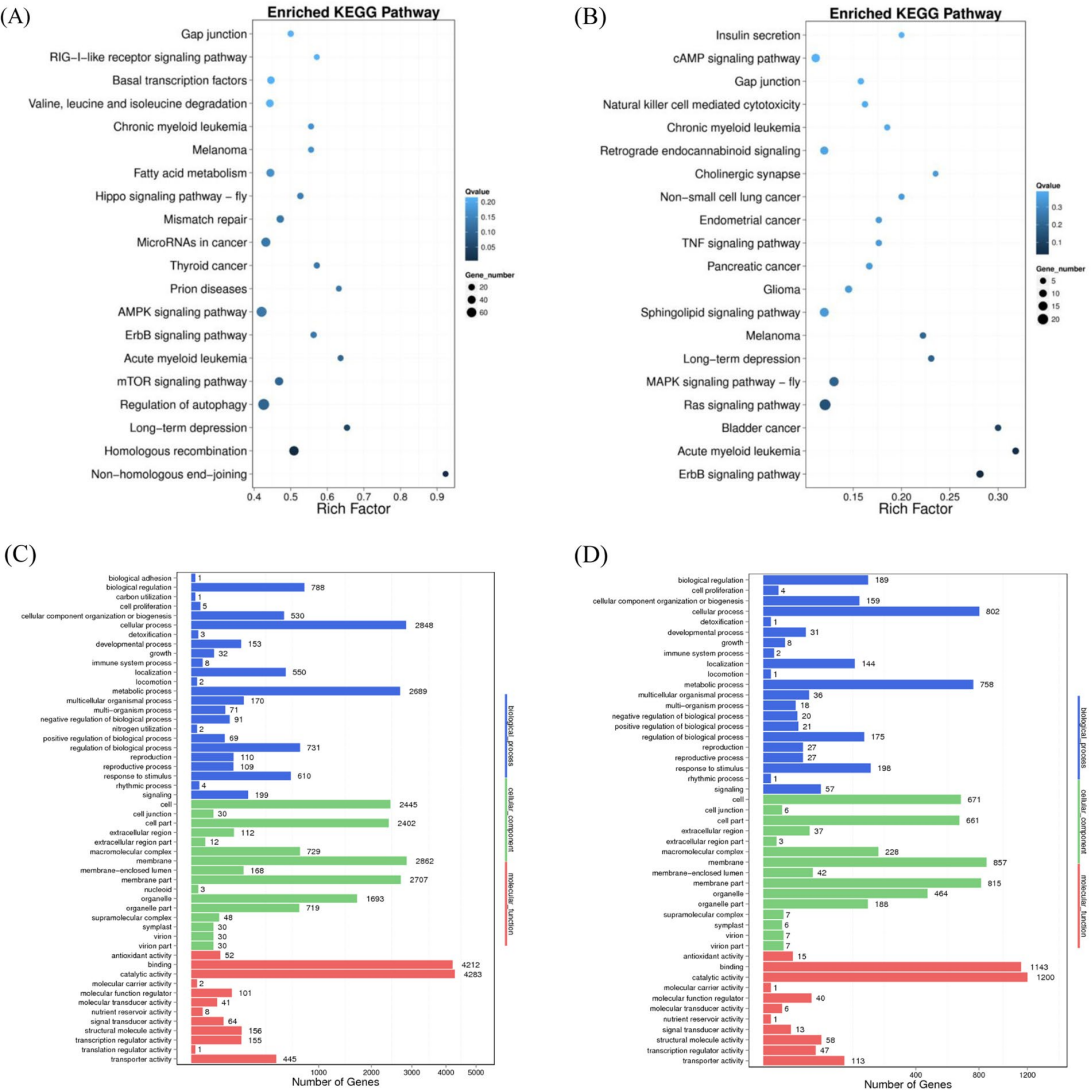
KEGG pathway enrichment and GO analysis of DMGs and DMPs which located in DMRs. (**A**) KEGG pathway enrichment of DMGs and (**B**) KEGG pathway enrichment of DMPs is shown. The size of each circle represents gene numbers, and the color represents the q-value. (**C**) GO analysis of DMGs and (**D**) GO analysis of DMPs is shown. The x-axis represents three domains of GO while y-axis represents the gene numbers within each pathway and processes.

Relative to control plants, 11,676 differentially methylated genes (DMGs) located in DMRs were identified (Table S5), and DMGs presented high methylation levels in mCG contexts with 11,020 DMGs, and low methylation levels in mCHG and mCHH contexts with 2,250 and 1,098 DMGs, respectively. These included 6,346 hypermethylated DMGs and 5,330 hypomethylated DMGs after drought stress, which indicated that some DMGs were also dynamically changed as a result of exposure to environmental stress. In order to investigate the biological functions of DMGs, a KEGG (Kyoto Encyclopedia of Genes and Genomes, http://www.genome.jp/kegg/) pathway analysis was performed. As shown in Fig. 5A, DMGs were mainly assigned to pathways related to the regulation of autophagy, AMPK signaling, homologous recombination, etc, which may be closely involved in the response to drought.

The promoter regions of genes affect transcriptional regulation, and differentially methylated promoters may affect transcriptional expression. To characterize changes in the methylation status of promoter regions in mulberry under drought stress, we analysed differentially methylated promoters (DMPs) located within DMRs and 4,233 DMPs were identified (Table S6), and DMPs presented highest methylation levels in mCG contexts with 2,550 DMGs, higher methylation levels in mCHH contexts with 1,502 DMPs and low methylation levels in mCHG contexts with 973 DMPs. These included 2,013 hypermethylated DMPs and 2,221 hypomethylated DMPs after drought stress. KEGG pathway analyses showed that DMPs were involved in the ras signaling pathway, MAPK signaling pathway, sphingolipid, and cAMP signaling pathways, etc (Fig. 5B). Many DMPs were involved in signaling pathways of some kind. In addition, methylated genes were highly enriched for the GO subcategories corresponding to catalytic activity, binding, membrane, cellular process, metabolic process, response to stimulus and regulation of biological process, suggesting that methylation mediates these types of pathways in mulberry (Figs. 5C,D).

### Correlation between DNA methylation and gene expression under drought stress

**Figure 6:**
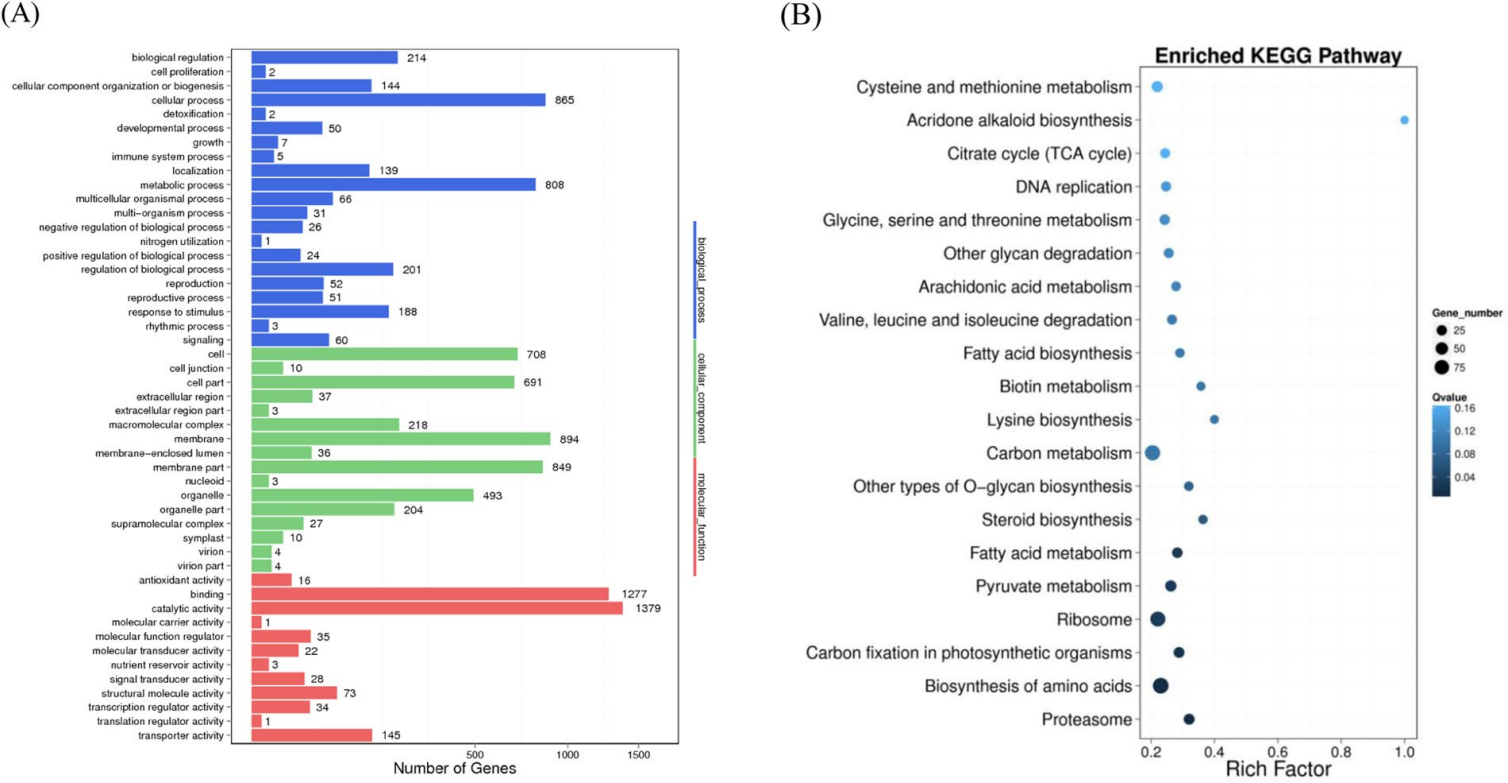
Distribution and functional enrichment analysis of significant difference in DMEGs. (**A**) GO analysis of DMEGs is shown. The x-axis represents three domains of GO while y-axis represents the gene numbers within each pathway and processes. (**B**) KEGG pathway enrichment of DMeGs is shown. The size of each circle represents gene numbers, and the color represents the q-value.

DNA methylation regulates genes in a wide variety of biological processes. To investigate potential transcriptional consequences of widespread methylation changes associated with drought stress, we performed mRNA-seq on the same normal growing conditioned and drought stressed mulberry plants that were used for methylation profiling. In total, 16010 different expression genes (DEGs) were identified between CK and DS (Table S7), and 5,751 and 10,259 DEGs were up-regulated and down-regulated, respectively. In order to explore whether the changes in mC methylation observed during drought stress altered gene expression, we synthetically analysed the data of DEGs and DMGs. As a result, we identified 3,243 different methylation and expression associated genes (DMEGs) that had a significant difference in methylation and expression levels in mulberry under drought stress compared to normal growing conditions (Table S8). To be specific, a total of 1,723 hypermethylated genes with up-regulated expression levels and 3,084 genes were hypermethylated with down-regulated expression levels between CK and DS. However, 2,631 hypomethylated genes were positively correlated with expression changes and 1,372 hypomethylated genes were negatively correlated with expression changes, which indicated a strong correlation between DNA methylation and gene expression. Moreover, GO analysis showed that DMEGs were highly enriched in catalytic activity, binding, membrane, cellular process and metabolic process, etc. (Fig. 6A). Further, related genes with great changes in methylation levels or methylation patterns may be closely linked to the drought stress response in mulberry. KEGG pathway analysis revealed that DMEGs were involved in pathways of carbon metabolism, ribosome, biosynthesis of amino acids, and cysteine and methionine metabolism, etc. (Fig. 6B), suggesting that the alteration of DNA methylation could regulate the expression of genes involved in multiple important pathways during drought stress in mulberry.

**Figure 7:**
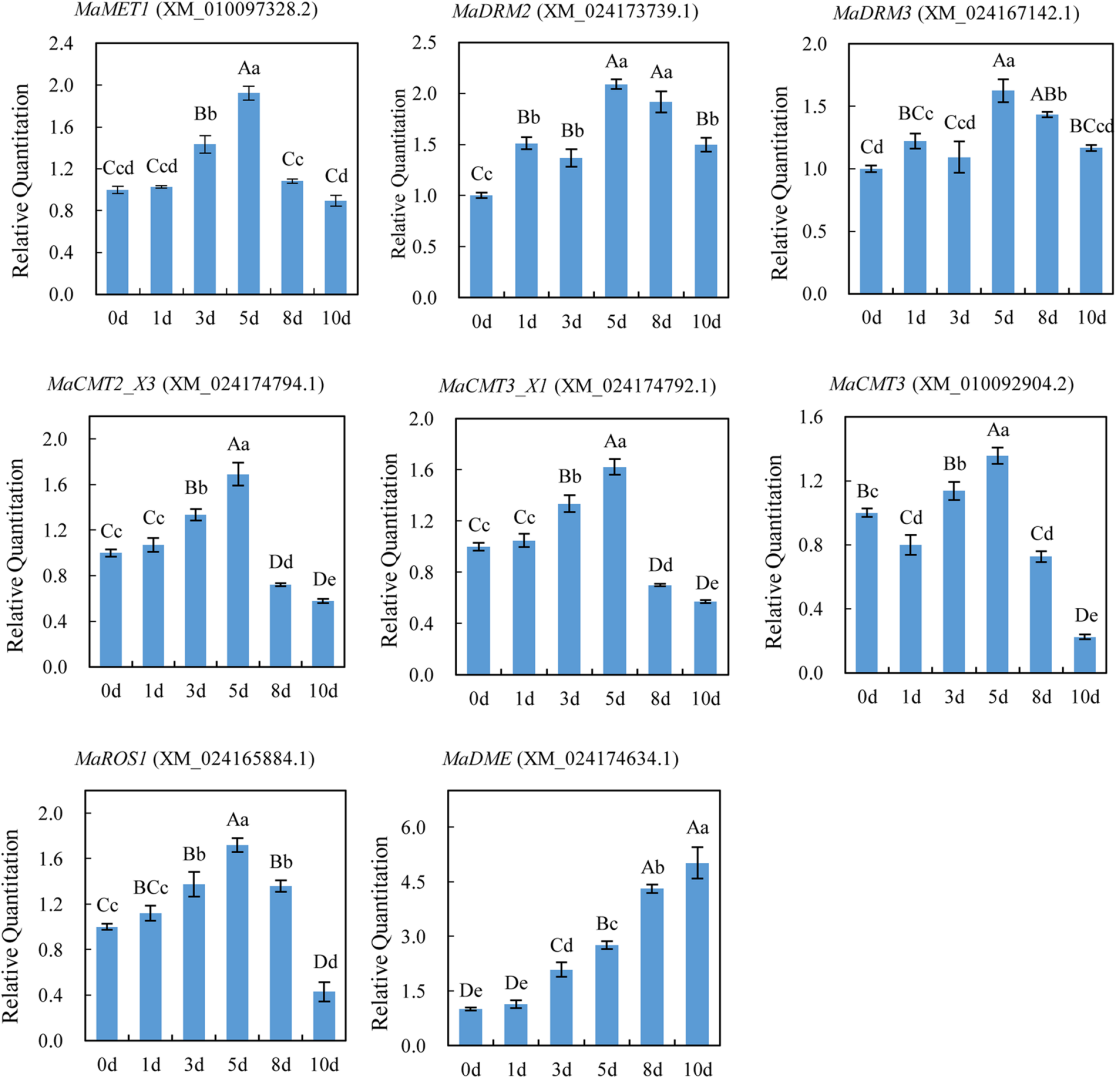
Relative expression analysis of genes encoding homologs of DNA methyltransferase and demethylases under drought stress for 0, 1, 3, 5, 8 and 10 days using qRT-PCR. β-actin was used as a normalisation control. The x axis represents different genes, and the y axis represents the relative expression level of genes. The results are mean ± SD of the triplicates of three biological replicates. Letter superscripts above bar indicate the significant difference of the changes between different drought stress time with the level of *P* > 0.05 (shown as the same or no letter superscripts), 0.01 < *P* ≤ 0.05 (shown as different small letter superscripts) and *P* ≤ 0.01 (shown as different capital letter superscripts).

At the genome scale, we globally investigated DNA methyltransferases and demethylases encoded by methylation-related genes in mulberry, which play crucial roles in maintenance of genomic methylation (Table S9). We found one MET1 (XP_010095630.1), and one DRM2 counterpart (XP_024029507.1) and one probable inactive DRM3 protein (XP_024022910.1) in mulberry genome. For CMT family, there were one CMT2 isoform (XP_024030562.1), one CMT3 (XP_010091206.1) and two CMT3 isoforms (XP_024030560.1, XP_024030561.1) in mulberry genome. Further, we analysed the expression profile of these genes in “Wansang 1” under drought stress for 0, 1, 3, 5, 8 and 10 days (Fig. 7). We can see that the expression levels of *MaMET1* under drought stress gradually ascend to the maximum with 1.93 fold (*P* ≤ 0.01) of the 0 day on the 5 day and then straightly reduced to the level of about 0.89 times (*P* ≤ 0.01) of the 0 day on the 10 day. The expression levels of *MaDRM* members were similar and got the highest at 5 days. *MaCMT2_X3* and *MaCMT3_X1* had the semblable express change and got the highest expression level at 5 days and presented the lowest level of about 0.57 times (*P* ≤ 0.01) of the 0 day at 10 days. The *MaCMT3* and putative *MaROS1* also showed highest expression level at 5 days. However, the *MaDME* expression gradually increased and went up to the maximum with 5.02 fold (*P* ≤ 0.01) of the 0 day on the 10 day. These results showed that drought stress would affect the expression pattern of genes encoding DNA methyltransferases and demethylases in mulberry.

### Methylation profiles of transposable elements in mulberry

**Figure 8:**
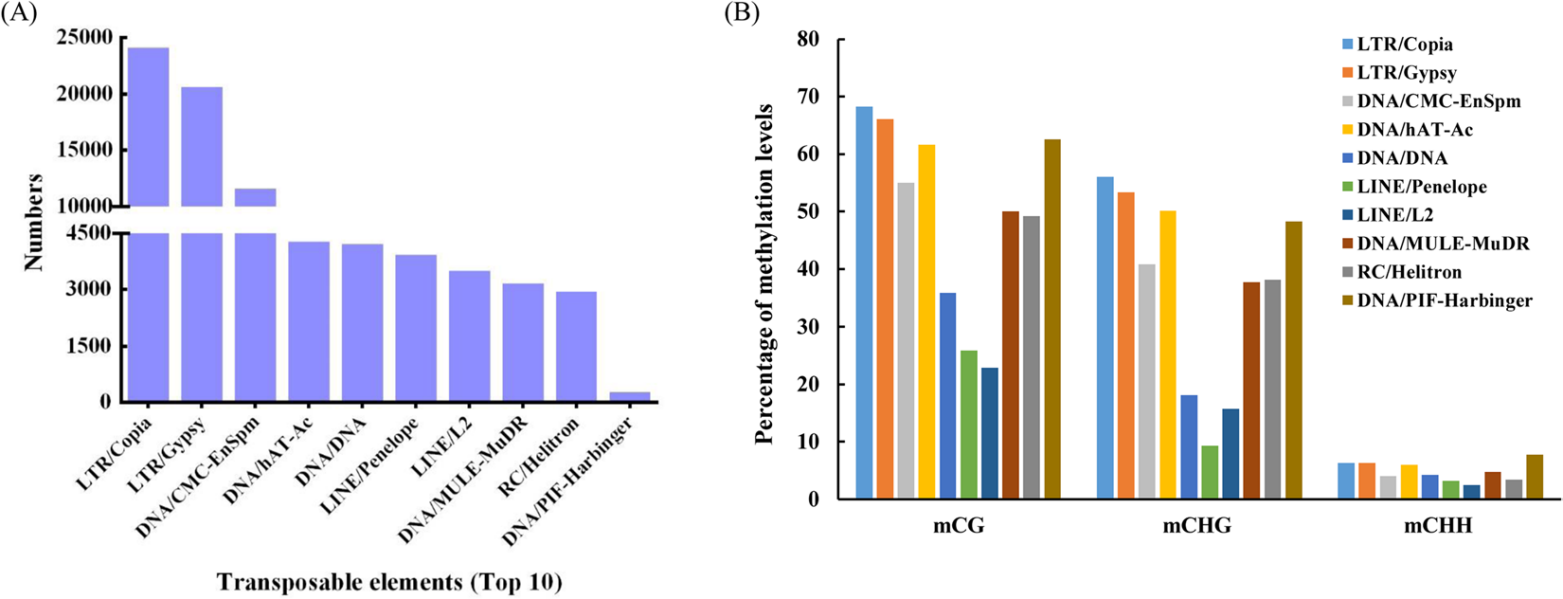
Methylation patterns of transposable elements in the mulberry genome. (**A**) Numbers of different types of TEs in mulberry genome. (**B**) Percentage of methylation levels of different types of TEs in mulberry.

Transposable elements (TEs) can affect genome size, create mutations with insertion and excision, and affect gene expression. To characterize the methylation profiles of transposable elements in mulberry, we first identified members of TEs and members of the top 10 various classes of TEs in the mulberry genome (Fig. 8A). We found 111,879 TEs in total and among these, Copia and Gypsy LTR-retrotransposons were the most numerous (24,146 LTR-Copia and 20,610 LTR-Gypsy). Then, the methylation level of these TEs was analysed. TEs presented high methylation levels in mCG and mCHG contexts, and different types of TEs showed similar mCG and mCHH methylation levels and with only minor differences in mCHG methylation (Fig. 8B). Moreover, to further characterize the methylation alterations of TEs under drought stress, we analysed methylation level of TEs between CK and DS. In total, 2,505 differentially methylated TEs (DMTEs) were identified between CK and DS, and there were 1,197, 1,145 and 791 DMTEs in mCG, mCHG, mCHH contexts, respectively. The majority of the differentially methylated TEs were methylated under drought stress, especially in mCHH contexts (Table S10). Furthermore, 673, 672 and 547 TEs were hypermethylated in mCG, mCHG and mCHH contexts, respectively, while 524, 473 and 244 TEs were hypomethylated. The results revealed that a large proportion of differentially TEs showed methylation in mCG, mCHG and mCHH contexts.

## Discussion

As a specifically operative technology, WGBS has been used for ascertaining methylation patterns with singlenucleotide resolution and has been used to decipher many plant methylomes, ranging from herbaceous plants like *Arabidopsis*^13^, rice^36^, tomato^37^ to woody plant like apple^30^, poplar^38^ and spruce^39^. Here, we deciphered the mulberry methylome by WGBS and found that CK-treated genome had methylation levels of ~9.95% (mCs), 33.03% (mCG), 20.63% (mCHG) and 2.86% (mCHH) for total sequenced C, CG, CHG and CHH sites, respectively, which reflected the percentage of methylation levels in the genome. Previous studies showed that mCG methylation shows the highest levels among the various species, ranging from ~30.5% in *Arabidopsis* to ~92.5% in *Beta vulgaris*. mCHG methylation varied from ~9.3% in *Eutrema salsugineum* to ~81.2% in *Beta vulgaris*, and mCHH methylation ranged from ~1.1% in *Vitis vinifera* to ~18.8% in *Beta vulgaris*^13,30,36,40^. Among various species, mulberry had moderate methylation levels. Based on previous studies, genomic DNA methylation patterns will be changed in response to drought stress^30,31,38^. Accordingly, DS-treated plants had methylation of ~10.81%, 35.09%, 20.96% and 2.98% in C, CG, CHG and CHH contexts showing that methylation levels were generally up-regulated after drought stress compared with controls. We also investigated the relative proportions of mCG, mCHG and mCHH of total mC sites in mulberry, presenting ~44.28%, ~28.50% and ~27.22% methylation levels at mCG, mCHG and mCHH sequences in CK mulberry plants. And our studies were consistent with the previous methylome researches which indicated mCG methylation was highest levels among three types of methylation, and mCHG and mCHH methylation levels tended to be lower^40^. Notably, drought stress was associated with a slight increases in the proportions of mCG and mCHG methylation, and a decrease in mCHH methylation in mulberry. These data showed that drought stress induced methylation in two sequence contexts, including mCpG and mCHG, and displayed hyper-methylation pattern after drought stress, which was consistent with previous research^41^.

Sub-telomeric regions of the chromosomes had greater DNA methylation densities, which might be important for the control of telomere length and recombination. Furthermore, the methylation status of mCG, mCHG and mCHH varied according to the age, location and physiology of a single organism^42^. In the mulberry genome, most of the mCG methylation sites were 80–100% methylated, while mCHG sites were 20–100% methylated and mCHH sites were 20–30% range. This trend was also observed in the genomic DNA of mulberry. Similar trends were also observed in *Populus trichocarpa* (percentages of mCG, mCHG and mCHH methylation were 41.9%, 20.9% and 3.25%, respectively)^43^ and *Betula platyphylla* (percentages of mCG, mCHG and mCHH were 42.6%, 28.8% and 5.2%, respectively)^44^. Work in *Arabidopsis thaliana*, however, revealed that the percentage of methylation of mCG, mCHG and mCHH were 24.60%, 6.98% and 1.70%, respectively^45^. These findings suggested that methylation levels dynamically change in accordance with external environmental conditions and are predominantly correlated with environmental factors.

DNA methylation status varied depending on the genomic region considered, which include euchromatin and heterochromatin, repetitive sequences and coding sequences. In general, mCG methylation is observed within both genes and repetitive sequences, and is involved in the regulation of gene expression^32,46^. Other than CG methylation, mCHG and mCHH types of methylation typically do not occur within genes and are mainly found in intergenic and repeat-rich regions of the genome. Methylation within these sequence contexts plays a critical role in the silencing of transposons^47^. mCG methylation was highest in TTRs and down2k regions from genes and lowest in the CDSs and exons. mCHG and mCHH methylation was highest within CpG islands and lowest in the CDSs and exons. Publications have previously revealed an obvious enrichment of methylation in TEs, and 5′-UTR regions had the lowest levels of methylation in *Betula platyphylla*^44^. mCG methylation accounted for approximately half of the total mC observed, and mCHH occurred least frequently in each genomic region and under both treatment (CK and DS), which suggested that mCG sites are the most important type among the three sequence contexts examined. This was in accordance with previous findings in upland cotton^31^. Repeat sequences were the most highly methylated among genomic regions assessed. In maize seedlings, methylation most frequently occurred in CpG islands within the 5′ regulatory regions of genes (promoters)^48^. To reveal the relationship between DNA methylation profiles and genes expression levels, canonical DNA methylation profiles of the entire transcriptional units were also divided into seven distinct functional elements in castor bean seeds^49,50^. The methylation level of the gene region and its adjacent bases are one of the epigenetic regulation mechanisms of gene expression. The distribution of the mCG methylation sites in 7 distinct genomic functional elements showed strongest regularity, while mCHG and mCHH methylation sites had the some regularity.

In some eukaryotic organisms, the occurrence of cytosine methylation has been associated with its nearest sequence context^13,31^. In the mulberry genome, the sequence preference around methylated sites were differed depending on CG and non-CG context for all methylated mC sites, and most methylation at mC sites occurred within the CAN (N represents A, T, C, G) context. There was no significant difference between normally grown mulberry plants and those exposed to drought stress. Study in *Arabidopsis thaliana* suggested that mCG methylation sites were highly enriched for the sequence ACGT, and methylation of mCHG sites were depleted of upstream cytosines, but tended to contain cytosine following the methylated base^13^. However, there has been no obvious sequence context specificity and no correlation between sequence context and methylation preference observed in white birch^44^.

DMRs are used for genetic imprinting because they can be methylated in accordance with either the maternal or the paternal chromosome. The methylated allele is often, but not always, the silenced allele. Differences between the methylation patterns of parental and offspring chromosomes are considered epigenetic lesions. In total, 20,485 DMRs were identified from plants subjected to drought stress. Relative to control plants, 11,676 DMGs and 4233 DMPs located in DMRs were identified, and they were mainly assigned to pathways related to the regulation of autophagy, AMPK signaling, homologous recombination, the ras signaling pathway, MAPK signaling pathway, sphingolipid and so on. Previous research has prompted scientists to hypothesise that gene body methylation suppresses spurious transcription from cryptic promoters that might otherwise interfere with the regulation of gene expression^43,51^, however, the function of gene body methylation requires further study^52^.

Under some certain circumstances, such as abiotic and biotic stresses, gene transcription level could be altered by the changes of DNA methylation, and lead to visible phenotypes. Many studies had shown that DNA methylation played an important role in the regulation of gene expression in response to various abiotic and biotic stresses in plants including *Arabidopsis*, dandelions, maize and *Pyropiahaitanensis*^53–56^. We identified 3243 different methylation and expression associated genes (DMEGs) that had a significant difference in methylation and expression levels in mulberry under drought stress compared to normal growing conditions, which demonstrated that there were obvious correlation between DNA methylation and gene transcription. The DMEGs were highly enriched in catalytic activity, binding, ribosome, biosynthesis of amino acids, and starch and sucrose metabolism. In this study, we found that many differentially DNA methylated regions could be induced by drought stress in mulberry plants, and some DNA methylomes regions were associated with gene expression. Similar results were also observed when *Arabidopsis*^57^ under pathogen infection and apple^30^ exposed to drought stress. The gene expression pattern could be affected by the changes of DNA methylation level through direct and indirect ways. In apple, the expression levels of methylation-related genes encoding DNA methyltransferases and demethylases had changed under water deficit^30^. The mRNA level of some methyltransferase and demethylase genes also had significant differences under drought stress in mulberry. These data will be useful for determining the impacts of differentially methylated regions on gene expression, transgenerational inheritance and stress response, and providing a means to explore the basis of resistance to facilitate mulberry breeding.

## Conclusions

In conclusion, we provide a high-resolution and high-coverage map that shows the methylation status of individual cytosines throughout the mulberry genome in response to drought stress. We found that the overall methylation level of plants subjected to DS was 8.64% higher than that of control plants, which was mainly due to the contribution of mCG methylation sites (6.24%). Further, methylcytosine was most frequently observed at mCG sites (44.28%), and less frequently observed at mCHG and mCHH sequences (28.50% and 27.22%, respectively). We further analysed the distribution of methylation sites throughout genomic regions and concluded that methylation tended to occur most frequently in distinct genomic elements. Our results showed the drought stress response in mulberry was probably initiated by the methylation of coding sequences and by the methylation of some promoters. Additionally, there were 3,243 DMEGs that displayed significantly different methylation and expression levels in control mulberry plants compared to those subjected to drought stress. In addition, methylated genes were highly enriched for the GO subcategories of catalytic activity, binding, membrane, cellular process, metabolic process, response to stimulus and regulation of biological process, suggesting that methylation mediates these pathways in mulberry. These data will require additional experimental data to confirm their role in the response to drought. This is the first study to comprehensively present a methylome map of the mulberry genome and reveal the occurrence of widespread DNA methylation changes in response to drought stress. The map will facilitate future studies investigating potential linkages between DNA methylation and gene expression in plants challenged with abiotic and biotic stresses.

## Methods

### Plant materials and drought stress treatment

**Figure 9:**
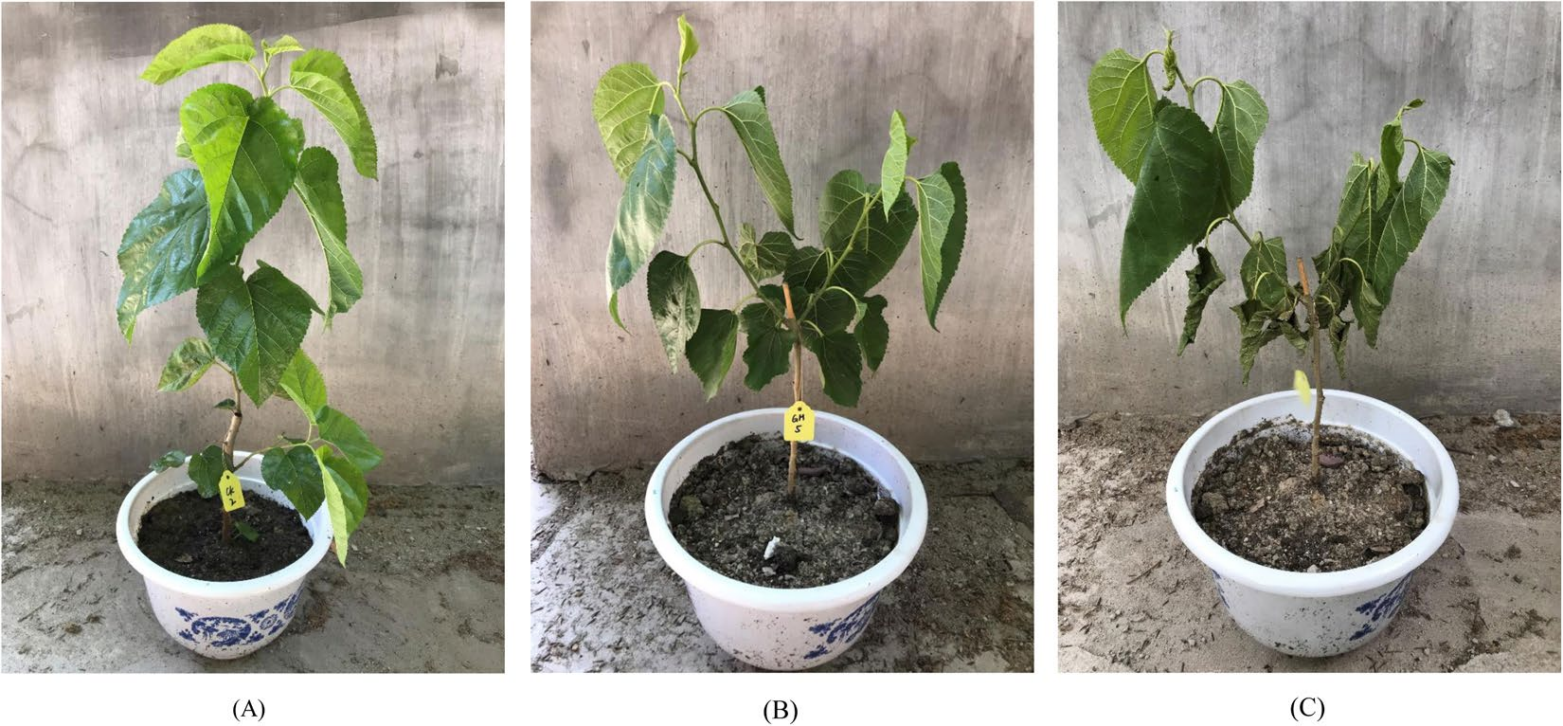
Effects of drought stress on phenotypic alterations of mulberry sapling. (**A**) Control mulberry sapling; (**B**) After drought treatment for 5 days; (**C**) After drought treatment for 10 days.

The mulberry species “Wansang 1” was obtained from the Anhui Mulberry Gene Bank in Hefei, Anhui, China. The plants were grown in the greenhouse under a photo period of 14 h light/10 h dark at 25 °C during the day and 20 °C at night, at 60–80% relative humidity according to previous study^58^. Mulberry cuttings were grafted to rootstocks. The grafted nurseries were planted in pots that were 35 cm in diameter that contained loam soil and one grafted plant per pot. The grafted plants were randomly grouped when new shoots had grown to 20 cm in length with two months old. The control group was well watered and irrigated daily to maintain 75–85% soil moisture that measured by soil moisture content analyser (WeikeMeituo, WKT-M3, China). The other group was exposed to drought stress for 5 d, 8 d and 10 d, and drought stress was conducted by withholding water to achieve 20–30% soil moisture (Fig. 9). Each group contained three replicates. At the end of treatments, young leaves were collected from the same position on each plant and immediately frozen in liquid nitrogen and stored at −80 °C for RNA extraction. The method used for sampling was the same for both the control and drought-stressed samples.

### DNA isolation, quantification and qualification

Total genomic DNA was extracted from leaves according to a modified CTAB DNA extraction protocol^59^. Genomic DNA quality and the presence of contamination were monitored by running samples on 1% agarose gels. DNA concentration was measured using Qubit DNA Assay Kit and a Qubit 2.0 Fluorometer (Life Technologies, USA). For the same treatment groups, equal quantities of DNA samples from different timepoints mixed together.

### Library construction and sequencing

The method of BS-seq library construction and sequencing in accordance with previous studies^30,31^. A total amount of 5.2 μg genomic DNA spiked with 26 ng lambda DNA was fragmented by sonication using a Bioruptor (Diagenode, Belgium) to a mean size of approximately 250 bp. This was followed by the formation of blunt ends in which dA addition to 3’-end, and adaptor ligation (in this case of methylated adaptors to protect from bisulfite conversion) was performed according to the manufacturer’s instructions. Lambda DNA was used as an unmethylated control for calculating the bisulphite conversion rate. Then, the ligated DNA was bisulfite converted using the EZ DNA Methylation-Gold kit (Zymo Research, USA). Different sized insert fragments were excised from the same lane of a 2% TAE agarose gel. Products were purified by using the QIAquick Gel Extraction kit (Qiagen, USA) according to the manufacturer’s protocol and amplified via PCR. BS-seq library constructions and sequencing was performed on three biological replicates per sample. Finally, libraries were sequenced using the Illumina Hi-Seq 2500 platform (BGI, Shenzhen, China). Three replicates from both control samples and drought stressed samples were assessed.

### Reads mapping to the reference genome

After sequencing data was delivered, we filtered results using SOAPnuke software (BGI, Shenzhen, China) to remove adaptor sequences, contamination and low-quality reads from raw reads. Low-quality reads included two types, and the reads meet anyone of the two conditions would be removed: 1) Unknown bases were more than 10%; 2) The ratio of bases whose quality was less than 20 was over 10%. After filtering, the remaining reads were called “clean reads” and the clean data was then mapped to the reference genome using BSMAP software^34^ using default parameters. The reference genome was converted into a bisulphite-converted version (conversion of C to T and G to A) and then indexed using Bowtie2^60^. Sequence reads were also transformed into fully bisulphite-converted versions (C to T and G to A converted) before reads were aligned to similarly converted versions of the genome in a directional manner. Then duplicated reads were removed and mapping results of each library were merged. Finally, we were able to calculate the mapping and bisulfite conversion rates for each sample.

### Analysis of methylation levels

Methylation levels (MLs) were determined by dividing the number of reads covering each mC site by the total reads covering that cytosine^61^, which was also equal to the mC/C ratio for each reference cytosine^32^. A sliding-window approach was applied for methylation level analyses. A window size of 3,000 bp and a step size of 600 bp^30^ was used. The sum of methylated and unmethylated read counts were calculated for each window. The methylation level of each C site reflected the fraction of methylated Cs observed, and was calculated using the following formula:

$${{\rm{ML}}}_{{\rm{average}}}{=[{\rm{Nm}}}_{{\rm{all}}}{/({\rm{Nm}}}_{{\rm{all}}}{+{\rm{Nnm}}}_{{\rm{all}}})]\times 100 \% $$

Nm represented the reads number of mC, while Nnm represented the reads number of nonmethylation reads.

Calculated ML was further corrected by considering the bisulfite nonconversion rate according to previous studies^62^. Given the bisulfite nonconversion rate r, the corrected ML was estimated using the following equationas:

$${{\rm{ML}}}_{{\rm{corrected}}}=({\rm{ML}}-{\rm{r}})/(1-{\rm{r}})$$

### Differentially methylated regions analysis

DMRs were identified using the swDMR software (http://122.228.158.106/ swDMR/), which used a sliding-window approach. The window was set to 1,000 bp and the step length was 100 bp. The Fisher test was implemented to detect DMRs^35^.

Putative DMRs were identified by comparing CK and DS methylomes using windows that contained at least 5 CpG (CHG or CHH) sites with a 2-fold change in MLs and Fisher test *p* value ≤ 0.05. In addition, we required that both tissues should not be hypomethylated when DMRs were identified. Two nearby DMRs were considered interdependent and were joined to form one continuous DMR if the genomic region from the start of an upstream DMR to the end of a downstream DMR also had 2-fold differences in MLs between CK and DS with *p* values ≤ 0.05. Otherwise, the two DMRs were viewed as independent. After iteratively merging interdependent DMRs, a final dataset containing independent DMRs was created.

To calculate the degree of difference between methyl-cytosines (mCG, mCHG, mCHH) of two samples while comparing MLs of DMRs from different samples CIRCOS was used according to the following formula^35^:

$${\rm{degree}}\,{\rm{of}}\,{\rm{difference}}={\log }_{2}{\rm{Rm}}1{/\log }_{2}{\rm{Rm}}2$$

Rm1, Rm2 represented the MLs of methyl-cytosine for CK and DS respectively, and 0.001 replaced Rm1 (or Rm2) in cases in which it equaled zero.

### RNA extraction and transcriptome sequencing

The mulberry plant materials used for transcriptome analysis were the same as the materials applied in methylome analysis and three biological replicates were used for transcriptome analysis. Total RNA was isolated using Trizol reagent (Invitrogen, USA) according to the instructions of the manufacturer. RNA quality and integrity were tested by Agilent Bioanalyzer 2100 (Agilent Technologies, Santa Clara, CA) and Nano Drop ND-1000 Spectrophotometer (Thermo Fisher Scientific, CA). A total amount of 1 μg qualified RNA was used for the RNA sample preparations and sequencing library was constructed using NEBNext Ultr RNA Library Prep Kit (NEB, Ipswich, MA) for Illumina according to the manufacturer’s instructions. The library fragments were purified and enriched with AMPure XP system (Beckman Coulter, Beverly, MA) to create final cDNA fragments with the size as 250–300 bp^28,30^. Finally, libraries were sequenced using the Illumina Hi-Seq 2500 platform (BGI, Shenzhen, China). Three replicates from both control samples and drought stressed samples were assessed.

### Identification of mulberry DMGs and qRT-PCR analysis

At the genome scale, we globally investigated DNA methyltransferases and demethylases encoded by methylation-related genes in mulberry, which play crucial roles in maintenance of genomic methylation, and analysed expression level of methylation-related genes under drought stress. “Wansang 1” mulberry plant materials were used for qRT-PCR analysis. Plants were grown in plastic pots, and were provided the same weight of soil and irrigation conditions. One-month-old, uniformly-grown plants were subjected to drought, and leaves were harvested after 0, 1, 3, 5, 8 and 10 days. The untreated samples (watered and irrigated daily) were used as the controls. Total RNA was isolated from the leaves of control and drought-stressed plants using RNAiso plus (Takara, China) according to the manufacturers’ instructions^58^. A total of 1.0 μg RNA was used for first-strand complementary DNA (cDNA) synthesis using M-MLV-reverse transcriptase (RTase) (Takara, China) according to the manufacturer’s instructions. RNA and primers were mixed with DEPC-treated water up to 17 μl and incubated at 70 °C for 10 min. Then the samples were subjected to immediate cooling on ice for more than 2 min. Then, 5 μl 5× RT-Buffer, 1.25 μl 10 mM dNTP, 0.75 μl RNase Inhibitor and 1 μl M-MLV RTase were combined in a total reaction volume of 20 μl. The synthesis was performed at 42 °C for 60 min and the inactivation of the enzyme was achieved by maintaining a temperature of 72 °C for 15 min. The resultant cDNA was used as a template for qRT-PCR and the amplification mixture volume was 20 μl per reaction. The internal housekeeping gene, β-actin, was used as a normalisation control^63^. The primers for targets and β-actin were added to FastStart Universal SYBR Green Master Mix kit (Roche, USA) according to the manufacturers instructions, and 20 μl of the reaction mixture was added to each well. Reactions were performed in a LightCycler 96 Real-Time PCR System (Roche, USA) with thermal cycling parameters that included 95 °C for 600 s followed by 45 cycles of 95 °C for 10 s, 59 °C for 10 s and 70 °C for 10 s^64^. The sequences of primers are listed in Table S11. Three biological replicates and three technical replicates for each sample were assessed. The relative expression differences of mRNAs were calculated using the 2^−ΔΔCt^ method. Standard errors and standard deviations were calculated from replicates and significance was measured via an one-way ANOVA and Duncan’s multiple test at the level of 0.01 < *P* ≤ 0.05 and *P* ≤ 0.01, respectively.

### GO and KEGG enrichment analysis of DMGs, DMPs and DMEGs

DMGs, DMPs and DMEGs were analysed for GO analysis and KEGG enrichment. GO is an international standard gene functional classification system, offers a dynamic-updated controlled vocabulary, as well as a strictly defined concept to comprehensively describe properties of genes and their products in any organism. GO enrichment analysis provided all GO terms that significantly enriched in a list of DMR related genes, comparing to a genome background, and filter the DMR-related genes that correspond to specific biological functions. Firstly, all DMR-related genes were mapped to GO terms in the database (http://www.geneontology.org/), calculating gene numbers for every term, then used hypergeometric test to find significantly enriched GO terms in the input list of DMGs, DMPs and DMEGs, based on “GO:: TermFinder” (http://www.yeastgenome.org/help/analyse/go-term-finder), and the method used was described as follows^65,66^:

$${\rm{P}}=1-\mathop{\sum }\limits_{i=0}^{m-1}\frac{\left(\frac{M}{i}\right)\left(\frac{N-M}{n-i}\right)}{\left(\frac{N}{n}\right)}$$

N was the number of all genes with GO annotation; n was the number of DMR-related genesin N; M was the number of all genes that were annotated to certain GO terms; and m was the number of DMGs, DMPs and DMEGs in M.

The calculated *P* value was subjected to a Bonferroni Correction^67^, and the corrected *P* value ≤ 0.05 was used as a threshold. GO terms fulfilling this condition were defined as significantly enriched GO terms in DMGs, DMPs and DMEGs, and this analysis was able to identify the main biological function of them. Pathway-based analyses helped us to further understand the biological functions of genes.

KEGG analysis was used to perform pathway enrichment analysis of DMGs, DMPs and DMEGs^68^. This analysis identified significantly enriched metabolic pathways or signal transduction pathways in DMGs and DMPs comparing with the whole genome background. The formula used to calculate KEGG enrichment was the same as the one used to determine GO enrichment (Equation 4). Here, N represented the number of all genes with the KEGG annotation, n represented the number of DMGs, DMPs and DMEGs in N, M was the total number of annotated to specific pathways, and m was the number of DMGs and DMPs in M. KOBAS software was used to test the statistical significance of enrichment of DMGs, DMPs and DMEGs in KEGG pathways^69,70^.

### Statistical analysis

The study was performed independently three times and each result shown in the figures was expressed as the mean ± standard deviation (SD). Statistical analysis was performed using Excel 2013 software (Microsoft, Redmond, USA) and SPSS Statistics 19.0 software (SPSS Inc., Chicago, IL, USA) with One-Way ANOVA Duncan’s multiple range test at the level of 0.01 < *P* ≤ 0.05 and *P* ≤ 0.01. Letters superscripted above the bar indicate the significant difference of the changes between differently treated groups with the level of *P* > 0.05 (shown as the same or no letter superscripts), 0.01 < *P* ≤ 0.05 (shown as different small letter superscripts or *) and *P* ≤ 0.01 (shown as different capital letter superscripts or **).

## Supplementary information

Supplementary Information.
