# Supplementary material for: Whole genome bisulfite sequencing methylome analysis of mulberry (Morus alba) reveals epigenome modifications in response to drought stress
Source: Sci Rep. 2020 May 15;10:8013. doi: 10.1038/s41598-020-64975-5 (PMC7228953; doi:10.1038/s41598-020-64975-5)
Supplement: Supplementary file 1 — Supplementary Information. [file 41598_2020_64975_MOESM1_ESM.zip › Supplementary information_Revised/Supplementary information.docx]

**^[[1]](#footnote-1)^ Whole genome bisulfit sequencing methylome analysis of mulberry (*Morus alba*) reveals** **epigenome modifications in response to drought stress**

Ruixue Li^1,a^, Fei Hu ^2,a^, Bing Li ^1^, Ming Chen ^1^,Yuping Zhang ^1^, Tan Fan ^1^& Taichu Wang ^1,*^

**Table S1.** Data summaryof Bisulphite Sequencing (BS-Seq) reads for two mulberry samples consisting of three replicates each

| **Sample ID** | **Clean Reads Number** | **Clean Data Size(bp)** | **Clean Rate(%)** |
| --- | --- | --- | --- |
| CK1 | 69,846,568 | 10,476,985,200 | 88.25 |
| CK2 | 90,293,876 | 13,544,081,400 | 88.82 |
| CK3 | 82,396,506 | 12,359,475,900 | 89.01 |
| DS1 | 90,936,240 | 13,640,436,000 | 89.18 |
| DS2 | 73,026,990 | 10,954,048,500 | 87.29 |
| DS3 | 79,672,832 | 11,950,924,800 | 89.96 |

**Table S2.** Effective coverage of each chromosome in CK and DS.

| **Sample** | **Chromosome** | **C (%)** | **CG (%)** | **CHG (%)** | **CHH (%)** |
| --- | --- | --- | --- | --- | --- |
| CK | fakechr1 | 44.82 | 42.37 | 47.37 | 44.79 |
|  | fakechr2 | 43.32 | 40.48 | 45.34 | 43.45 |
|  | fakechr3 | 45.33 | 42.61 | 47.75 | 45.37 |
|  | fakechr4 | 45.12 | 42.53 | 47.65 | 45.12 |
|  | fakechr5 | 43.66 | 40.77 | 46.10 | 43.72 |
|  | fakechr6 | 45.25 | 42.51 | 47.67 | 45.29 |
|  | fakechr7 | 34.58 | 32.38 | 36.28 | 34.66 |
| DS | fakechr1 | 44.17 | 42.22 | 47.07 | 43.99 |
|  | fakechr2 | 42.59 | 40.27 | 45.03 | 42.55 |
|  | fakechr3 | 44.57 | 42.34 | 47.38 | 44.45 |
|  | fakechr4 | 44.34 | 42.27 | 47.26 | 44.18 |
|  | fakechr5 | 43.001 | 40.66 | 45.84 | 42.90 |
|  | fakechr6 | 44.49 | 42.24 | 47.27 | 44.37 |
|  | fakechr7 | 33.90 | 32.15 | 35.94 | 33.83 |

**Table S3.** Percentage of methylation levels of whole genome in CK and DS.

| **Sample** | **C (%)** | **CG (%)** | **CHG (%)** | **CHH (%)** |
| --- | --- | --- | --- | --- |
| CK1 | 9.90 | 32.73 | 20.47 | 2.95 |
| CK2 | 10.20 | 33.45 | 20.93 | 2.94 |
| CK3 | 9.74 | 32.92 | 20.47 | 2.69 |
| DS1 | 11.01 | 33.29 | 21.85 | 3.59 |
| DS2 | 10.94 | 34.64 | 22.35 | 3.22 |
| DS3 | 10.48 | 33.51 | 21.65 | 2.94 |

**Table S4.** Proportion of CG, CHG and CHH in all methyl-cytosine.

| **Sample** |  | **CG** | **CHG** | **CHH** |
| --- | --- | --- | --- | --- |
| CK1 | number | 1,467,004 | 938,296 | 804,537 |
|  | proportion (%) | 45.70 | 29.23 | 25.07 |
| CK2 | number | 1,500,110 | 990,194 | 1,076,438 |
|  | proportion (%) | 42.06 | 27.76 | 30.18 |
| CK3 | number | 1,366,239 | 864,357 | 800,632 |
|  | proportion (%) | 45.07 | 28.52 | 26.41 |
| DS1 | number | 1,425,108 | 920,573 | 830,982 |
|  | proportion (%) | 44.86 | 28.98 | 26.16 |
| DS2 | number | 1,519,906 | 984,697 | 922,955 |
|  | proportion (%) | 44.34 | 28.73 | 26.93 |
| DS3 | number | 1,409,617 | 913,195 | 803,380 |
|  | proportion (%) | 45.09 | 29.21 | 25.70 |

**Table S5.** The DMGs which located in DMRs.

**Table S6.** The DMPs which located in DMRs.

**Table S7.** The DEGs between CK and DS.

**Table S8.** The DMEGs (mCpG. DMR-mRNA).

**Table S9.** Homologs of DNA methyltransferase and demethylase proteins in mulberry.

| **Protein** | ***Morus notabilis* (GenBank)** | **Length (aa)** | **Location** | **Gene** | **Gene ID** |
| --- | --- | --- | --- | --- | --- |
| MET1 | XP_010095630.1 | 1557 | NW_010360769.1 (28756..35242) | XM_010097328.2 | 21387255 |
| CMT2_X3 | XP_024030562.1 | 1193 | NW_010367234.1 (470003..492596) | XM_024174794.1 | 21405623 |
| CMT3_X1 | XP_024030560.1 | 1392 | NW_010367234.1 (470003..492596) | XM_024174792.1 | 21405623 |
| CMT3_X2 | XP_024030561.1 | 1391 | NW_010367234.1 (470003..492596) | XM_024174793.1 | 21405623 |
| CMT3 | XP_010091206.1 | 851 | NW_010358644.1 (47210..52497) | XM_010092904.2 | 21389951 |
| DRM2 | XP_024029507.1 | 636 | NW_010366566.1 (273609..278107) | XM_024173739.1 | 112093987 |
| DRM3 | XP_024022910.1 | 703 | NW_010361652.1 (1031848..1038761) | XM_024167142.1 | 21408804 |
|  | XP_024022909.1 | 703 | NW_010361652.1 (1031848..1038761) | XM_024167141.1 | 21408804 |
| DME | XP_024030402.1 | 1931 | NW_010367100.1 (234172..245190) | XM_024174634.1 | 21399835 |
| ROS1 | XP_024021652.1 | 1640 | NW_010361124.1  (81774..90119) | XM_024165884.1 | 21387095 |

**Table S10.** The DMTEs between CK and DS.

**Table S11.** The primer sequences for qRT-PCR analysis of genes.

| Gene | **primer** | **sequence (5’-3’)** |
| --- | --- | --- |
| *MaMET1*  XM_010097328.2 | forward primer | TGACAAGGACAAGGAACGGG |
|  | reverse primer | GACAGCCATACGACAGGGAC |
| *MaCMT2_X3*  XM_024174794.1 | forward primer | TCCACTTCCAACCCACGATG |
|  | reverse primer | CACGAGGTTGGTCTTCGTCA |
| *MaCMT3_X1*  XM_024174792.1 | forward primer | CAAGCATTCCTCCCGTTCCT |
|  | reverse primer | GTGCTACCGGTGTGATGGAA |
| *MaCMT3*  XM_010092904.2 | forward primer | TGCAAGCTGAGTTTTGCGTT |
|  | reverse primer | TACTTTGTGGATCGGCCACC |
| *MaDRM2*  XM_024173739.1 | forward primer | ATTTGTTCGACCTCGCCGAT |
|  | reverse primer | ATGGGCTTCCACCAACTACG |
| *MaDRM3*  XM_024167142.1 | forward primer | GGAGCGGAAGGTATCCGAAG |
|  | reverse primer | AGCCTCTGTTCGGCATTGAA |
| *MaROS1*  XM_024165884.1 | forward primer | TCCAAGTTCGCAGGACCATC |
|  | reverse primer | TTGCTCTCGCGATAAGGGAC |
| *MaDME*  XM_024174634.1 | forward primer | ATCCAGATTTGGCTGCCTCC |
|  | reverse primer | ATGAGCTGCAATCTCCTCGG |
| β-actin | forward primer | TGGCTTATGTTGCCTTGGAC |
|  | reverse primer | GTTGGAAGAGGACTTGTGGG |

**Figure S1.** Distribution of cytosine methylation throughout chromosome 3.

**Figure S2.** Distribution of cytosine methylation throughout chromosome 4.

**Figure S3.** Distribution of cytosine methylation throughout chromosome 5.

**Figure S4.** Distribution of cytosine methylation throughout chromosome 6.

**Figure S5.** Distribution of cytosine methylation throughout chromosome 7.

**Figure S6.** Distribution of methylation levels within each sequence context. Only the mCs covered by at least four reads were used to calculate methylation levels. The x-axis was defined as the percentage of reads showing methylated cytosines at a reference cytosine site. The y-axis indicates the fraction of the total number of methyl cytosines calculated within bins of 10%.

1. ^1^Sericultural Research Institute, Anhui Academy of Agricultural Sciences, Hefei, Anhui, 230061, China. ^2^Plant Protection and Agroproducts Safety Institute, Anhui Academy of Agricultural Sciences, Hefei, Anhui, 230031, China. Correspondence and requests for materials should be addressed to T.W. (email: wangtaichu123@163.com). ^a^These authors contributed equally to this work. [↑](#footnote-ref-1)
