# Supplementary material for: Whole genome bisulfite sequencing methylome analysis of mulberry (Morus alba) reveals epigenome modifications in response to drought stress
Source: Sci Rep. 2020 May 15;10:8013. doi: 10.1038/s41598-020-64975-5 (PMC7228953; doi:10.1038/s41598-020-64975-5)
Supplement: Supplementary file 1 — Supplementary Information. [file 41598_2020_64975_MOESM1_ESM.zip › Supplementary information_Revised/Supplementary information.pdf]

Whole genome bisulfit sequencing methylome analysis of mulberry (*Morus alba*) reveals epigenome modifications in response to drought stress

Ruixue Li[1,a], Fei Hu[2,a], Bing Li[1], Ming Chen[1],Yuping Zhang[1], Tan Fan[1]& Taichu Wang[1,*]

[1]Sericultural Research Institute, Anhui Academy of Agricultural Sciences, Hefei, Anhui, 230061, China. [2]Plant Protection and Agroproducts Safety Institute, Anhui Academy of Agricultural Sciences, Hefei, Anhui, 230031, China. Correspondence and requests for materials should be addressed to T.W. (email: wangtaichu123@163.com). [a]These authors contributed equally to this work.

Table S1. Data summaryof Bisulphite Sequencing (BS-Seq) reads for two mulberry samples consisting of three replicates each

Sample ID	Clean Reads Number	Clean Data Size(bp)	Clean Rate(%)
CK1	69,846,568	10,476,985,200	88.25
CK2	90,293,876	13,544,081,400	88.82
CK3	82,396,506	12,359,475,900	89.01
DS1	90,936,240	13,640,436,000	89.18
DS2	73,026,990	10,954,048,500	87.29
DS3	79,672,832	11,950,924,800	89.96

Table S2. Effective coverage of each chromosome in CK and DS.

Sample	Chromosome	C (%)	CG (%)	CHG (%)	CHH (%)
CK	fakechr1	44.82	42.37	47.37	44.79
	fakechr2	43.32	40.48	45.34	43.45
	fakechr3	45.33	42.61	47.75	45.37
	fakechr4	45.12	42.53	47.65	45.12
	fakechr5	43.66	40.77	46.10	43.72
	fakechr6	45.25	42.51	47.67	45.29
	fakechr7	34.58	32.38	36.28	34.66
DS	fakechr1	44.17	42.22	47.07	43.99
	fakechr2	42.59	40.27	45.03	42.55
	fakechr3	44.57	42.34	47.38	44.45
	fakechr4	44.34	42.27	47.26	44.18
	fakechr5	43.001	40.66	45.84	42.90
	fakechr6	44.49	42.24	47.27	44.37
	fakechr7	33.90	32.15	35.94	33.83

Table S3. Percentage of methylation levels of whole genome in CK and DS.

Sample	C (%)	CG (%)	CHG (%)	CHH (%)
CK1	9.90	32.73	20.47	2.95
CK2	10.20	33.45	20.93	2.94
CK3	9.74	32.92	20.47	2.69
DS1	11.01	33.29	21.85	3.59
DS2	10.94	34.64	22.35	3.22
DS3	10.48	33.51	21.65	2.94

Table S4. Proportion of CG, CHG and CHH in all methyl-cytosine.

Sample		CG	CHG	CHH
CK1	number	1,467,004	938,296	804,537
	proportion (%)	45.70	29.23	25.07
CK2	number	1,500,110	990,194	1,076,438
	proportion (%)	42.06	27.76	30.18
CK3	number	1,366,239	864,357	800,632
	proportion (%)	45.07	28.52	26.41
DS1	number	1,425,108	920,573	830,982
	proportion (%)	44.86	28.98	26.16
DS2	number	1,519,906	984,697	922,955
	proportion (%)	44.34	28.73	26.93
DS3	number	1,409,617	913,195	803,380
	proportion (%)	45.09	29.21	25.70

Table S5. The DMGs which located in DMRs.

Table S6. The DMPs which located in DMRs.

Table S7. The DEGs between CK and DS.

Table S8. The DMEGs (mCpG. DMR-mRNA).

Table S9. Homologs of DNA methyltransferase and demethylase proteins in mulberry.

Protein	*Morus notabilis* (GenBank)	Length (aa)	Location	Gene	Gene ID
MET1	XP_010095630.1	1557	NW_010360769.1 (28756..35242)	XM_010097328.2	21387255
CMT2_X3	XP_024030562.1	1193	NW_010367234.1 (470003..492596)	XM_024174794.1	21405623
CMT3_X1	XP_024030560.1	1392	NW_010367234.1 (470003..492596)	XM_024174792.1	21405623
CMT3_X2	XP_024030561.1	1391	NW_010367234.1 (470003..492596)	XM_024174793.1	21405623
CMT3	XP_010091206.1	851	NW_010358644.1 (47210..52497)	XM_010092904.2	21389951
DRM2	XP_024029507.1	636	NW_010366566.1 (273609..278107)	XM_024173739.1	112093987
DRM3	XP_024022910.1	703	NW_010361652.1 (1031848..1038761)	XM_024167142.1	21408804
	XP_024022909.1	703	NW_010361652.1 (1031848..1038761)	XM_024167141.1	21408804
DME	XP_024030402.1	1931	NW_010367100.1 (234172..245190)	XM_024174634.1	21399835
ROS1	XP_024021652.1	1640	NW_010361124.1 (81774..90119)	XM_024165884.1	21387095

Table S10. The DMTEs between CK and DS.

Table S11. The primer sequences for qRT-PCR analysis of genes.

Gene	**primer**	**sequence (5'-3')**
MaMET1	forward primer	TGACAAGGACAAGGAACGGG
XM_010097328.2	reverse primer	GACAGCCATACGACAGGGAC
MaCMT2_X3	forward primer	TCCACTTCCAACCCACGATG
XM_024174794.1	reverse primer	CACGAGGTTGGTCTTCGTCA
MaCMT3_X1	forward primer	CAAGCATTCCTCCCGTTCCT
XM_024174792.1	reverse primer	GTGCTACCGGTGTGATGGAA
MaCMT3	forward primer	TGCAAGCTGAGTTTTGCGTT
XM_010092904.2	reverse primer	TACTTTGTGGATCGGCCACC
MaDRM2	forward primer	ATTTGTTCGACCTCGCCGAT
XM_024173739.1	reverse primer	ATGGGCTTCCACCAACTACG
MaDRM3	forward primer	GGAGCGGAAGGTATCCGAAG
XM_024167142.1	reverse primer	AGCCTCTGTTCGGCATTGAA
MaROS1	forward primer	TCCAAGTTCGCAGGACCATC
XM_024165884.1	reverse primer	TTGCTCTCGCGATAAGGGAC
MaDME	forward primer	ATCCAGATTTGGCTGCCTCC
XM_024174634.1	reverse primer	ATGAGCTGCAATCTCCTCGG
β-actin	forward primer	TGGCTTATGTTGCCTTGGAC
	reverse primer	GTTGGAAGAGGACTTGTGGG

Figure S1. Distribution of cytosine methylation throughout chromosome 3.

Figure S2. Distribution of cytosine methylation throughout chromosome 4.

Figure S3. Distribution of cytosine methylation throughout chromosome 5.

Figure S4. Distribution of cytosine methylation throughout chromosome 6.

Figure S5. Distribution of cytosine methylation throughout chromosome 7.

Figure S6. Distribution of methylation levels within each sequence context. Only the mCs covered by at least four reads were used to calculate methylation levels. The x-axis was defined as the percentage of reads showing methylated cytosines at a reference cytosine site. The y-axis indicates the fraction of the total number of methyl cytosines calculated within bins of 10%.
