# Supplementary figures and images for: Whole genome bisulfite sequencing methylome analysis of mulberry (Morus alba) reveals epigenome modifications in response to drought stress
Source: Sci Rep. 2020 May 15;10:8013. doi: 10.1038/s41598-020-64975-5 (PMC7228953; doi:10.1038/s41598-020-64975-5)

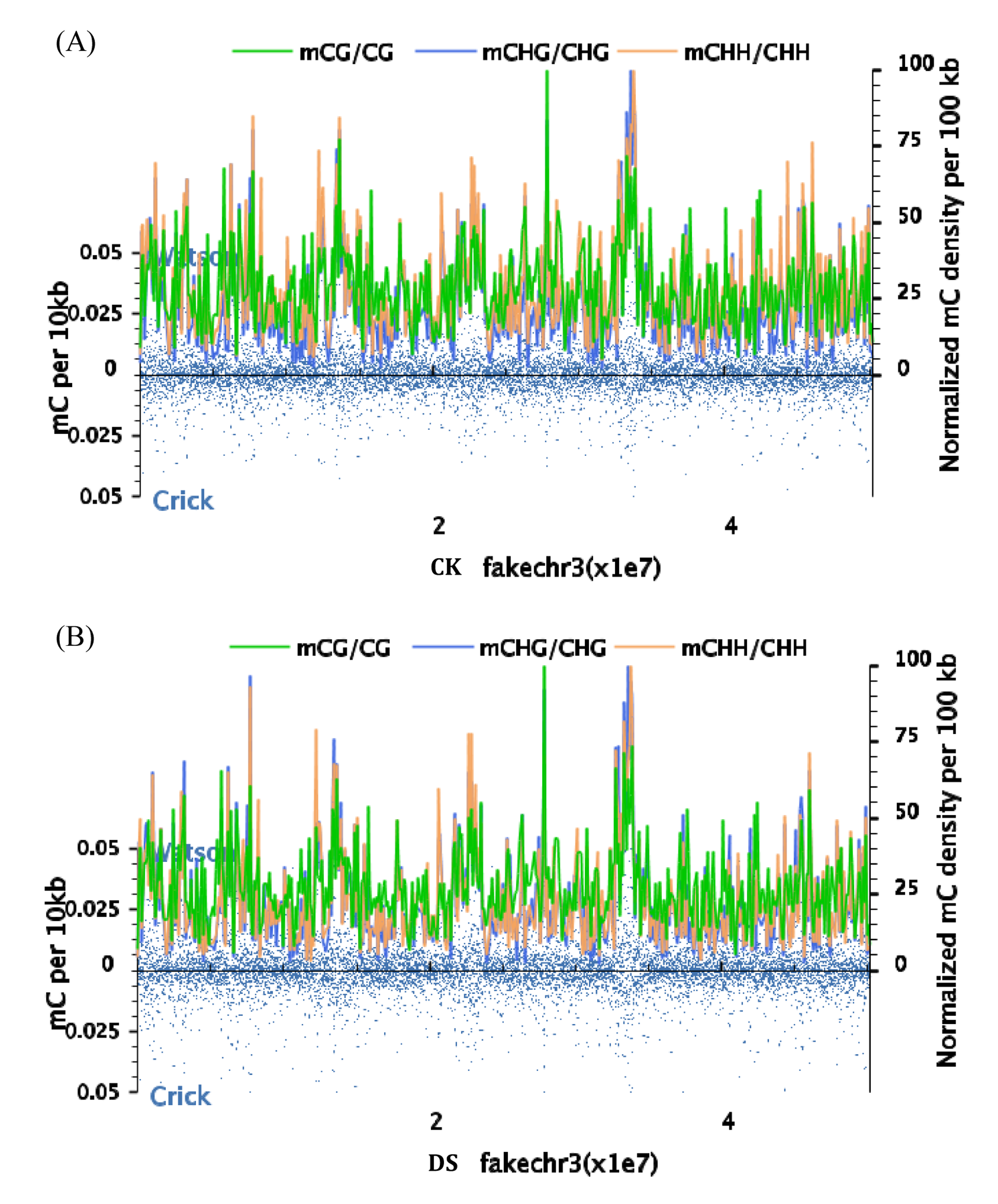

Supplement: Supplementary file 1 — Supplementary Information. [file 41598_2020_64975_MOESM1_ESM.zip › Supplementary information_Revised/FigureS1.tif]

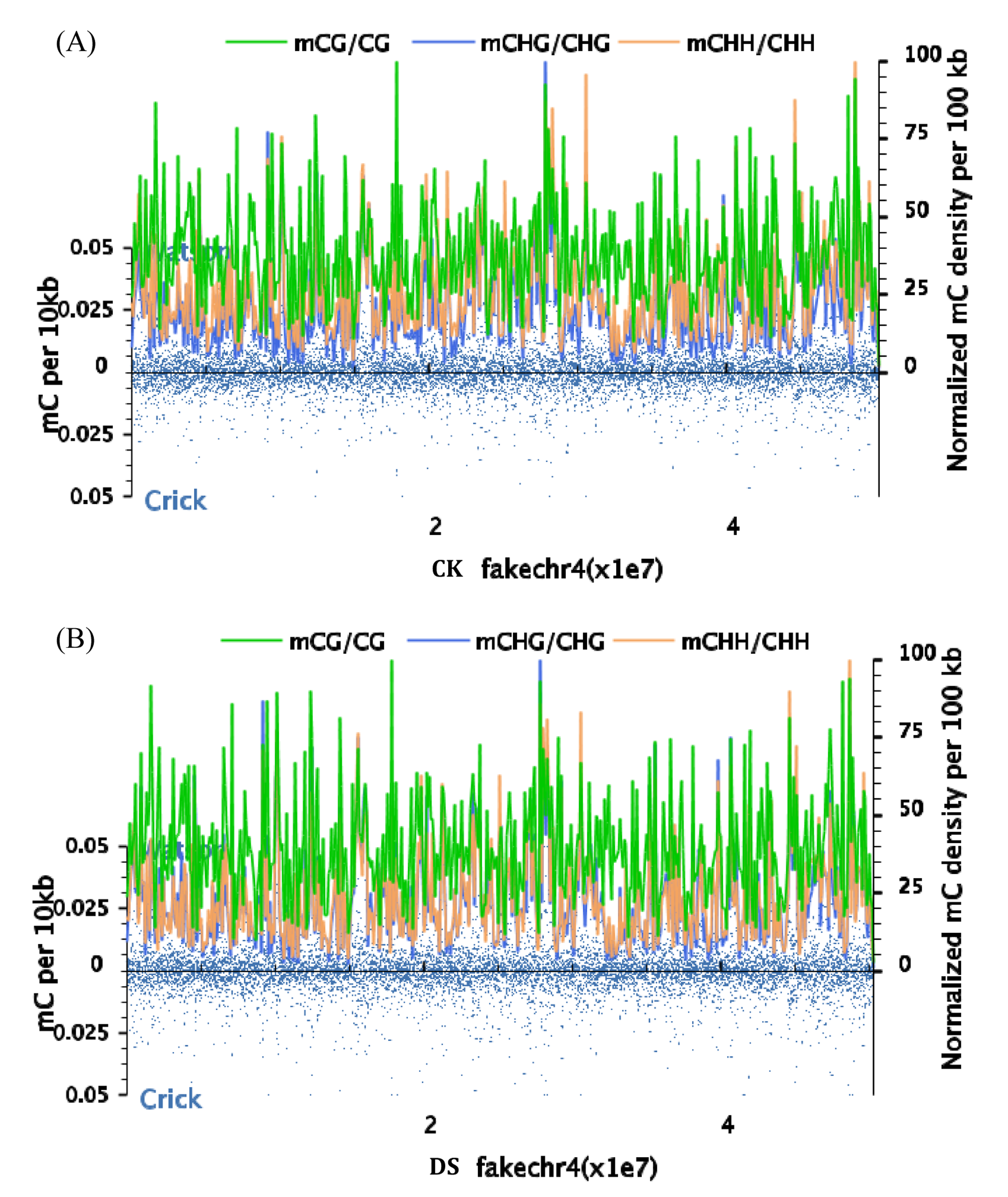

Supplement: Supplementary file 1 — Supplementary Information. [file 41598_2020_64975_MOESM1_ESM.zip › Supplementary information_Revised/FigureS2.tif]

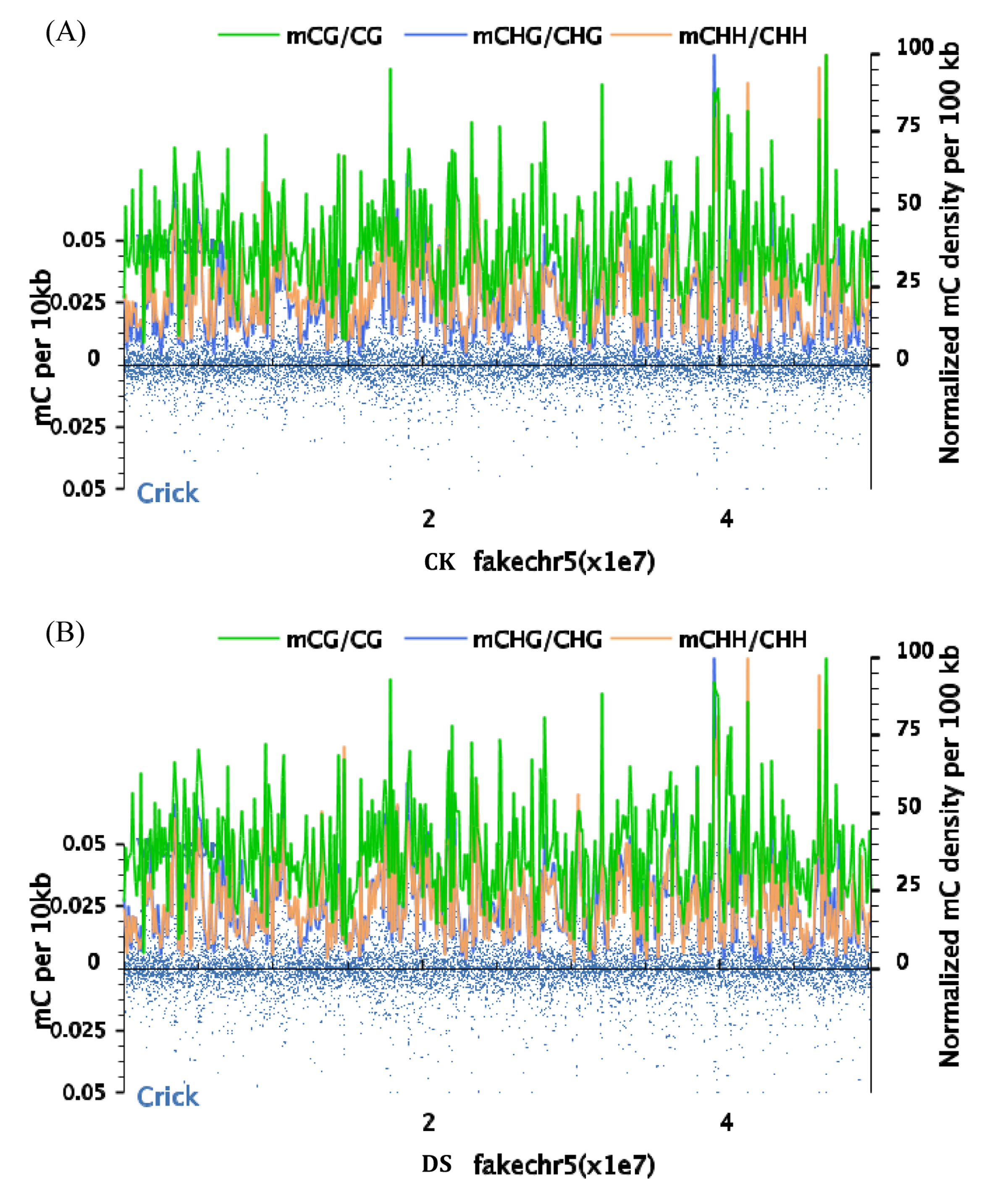

Supplement: Supplementary file 1 — Supplementary Information. [file 41598_2020_64975_MOESM1_ESM.zip › Supplementary information_Revised/FigureS3.tif]

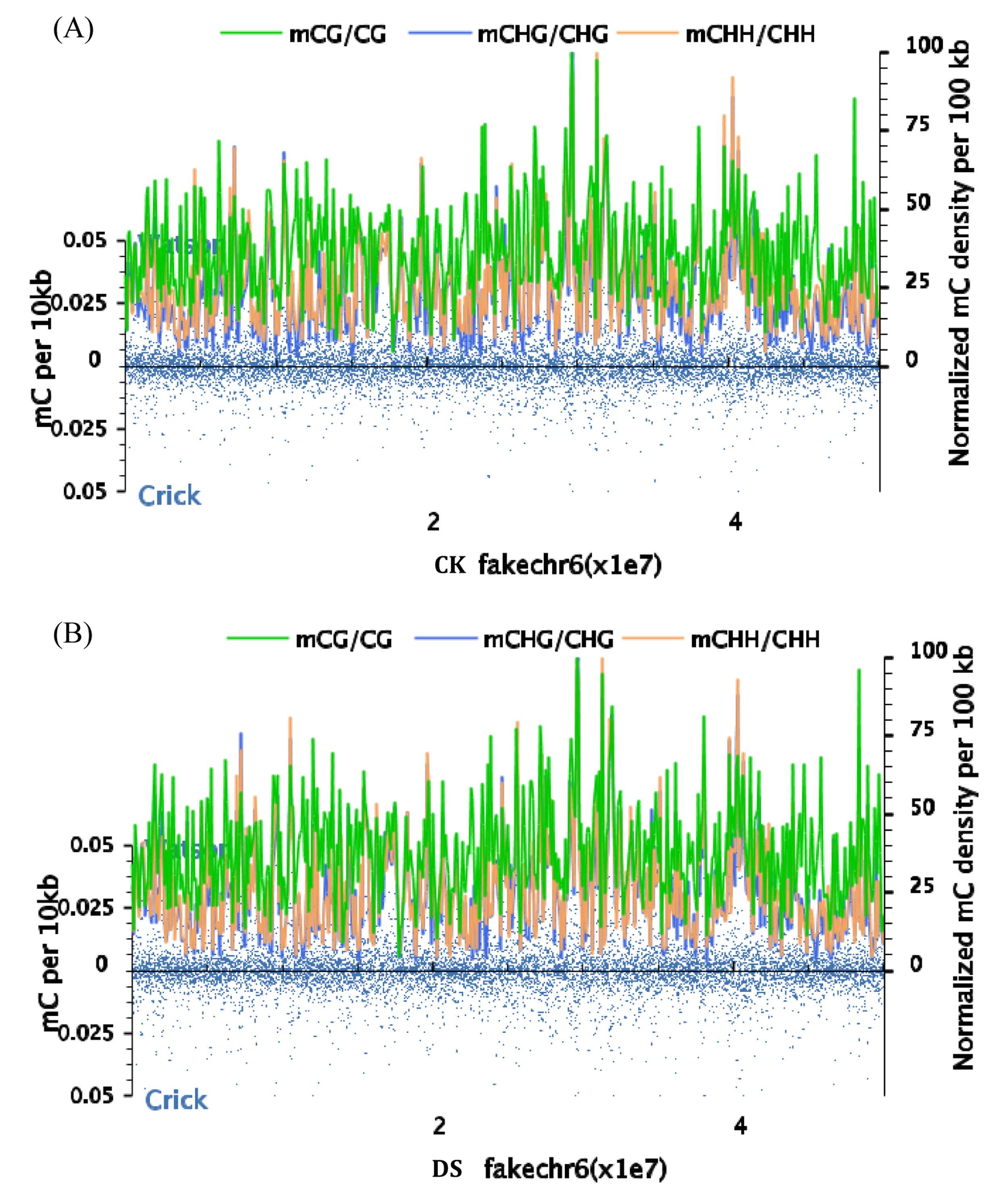

Supplement: Supplementary file 1 — Supplementary Information. [file 41598_2020_64975_MOESM1_ESM.zip › Supplementary information_Revised/FigureS4.tif]

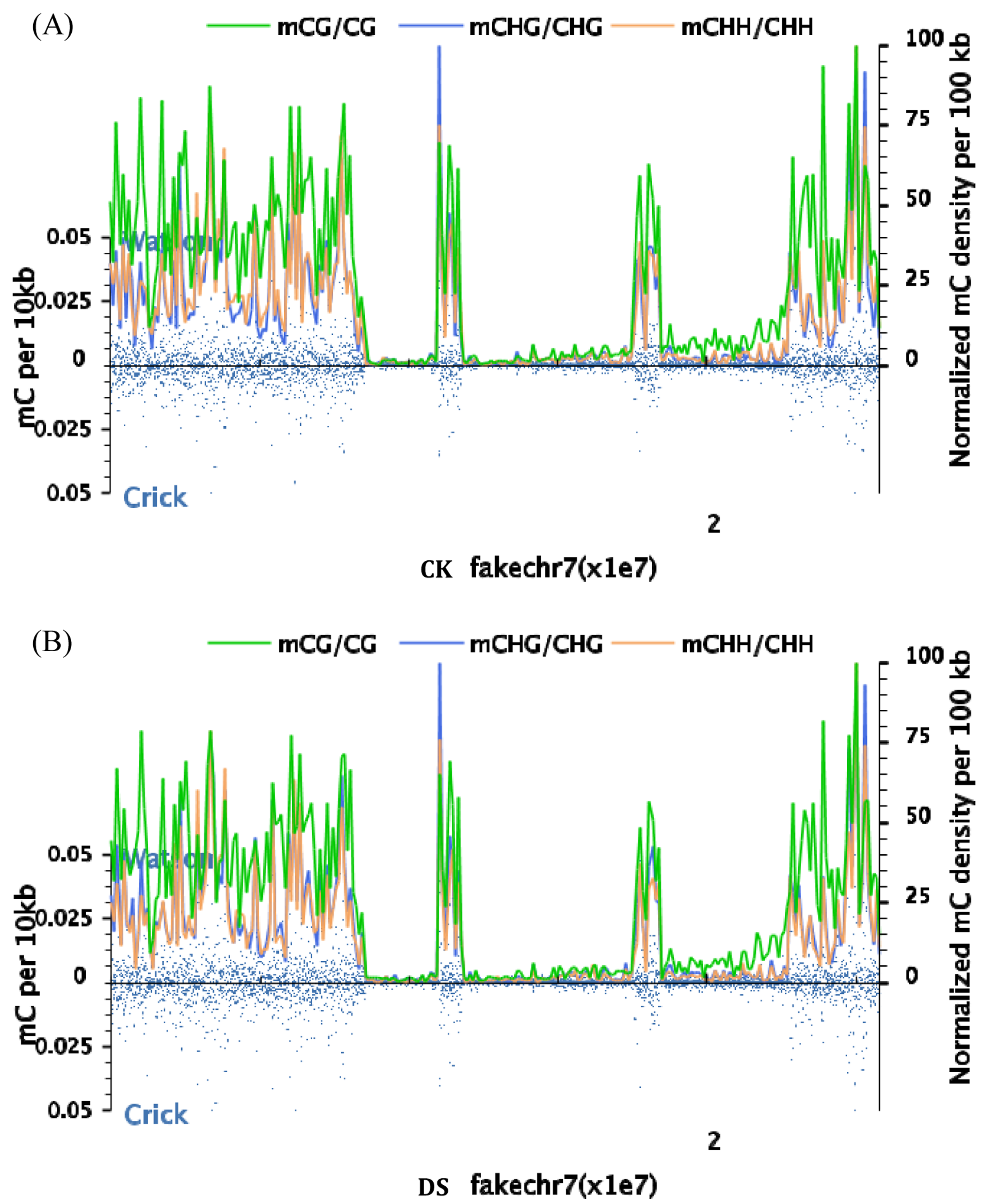

Supplement: Supplementary file 1 — Supplementary Information. [file 41598_2020_64975_MOESM1_ESM.zip › Supplementary information_Revised/FigureS5.tif]

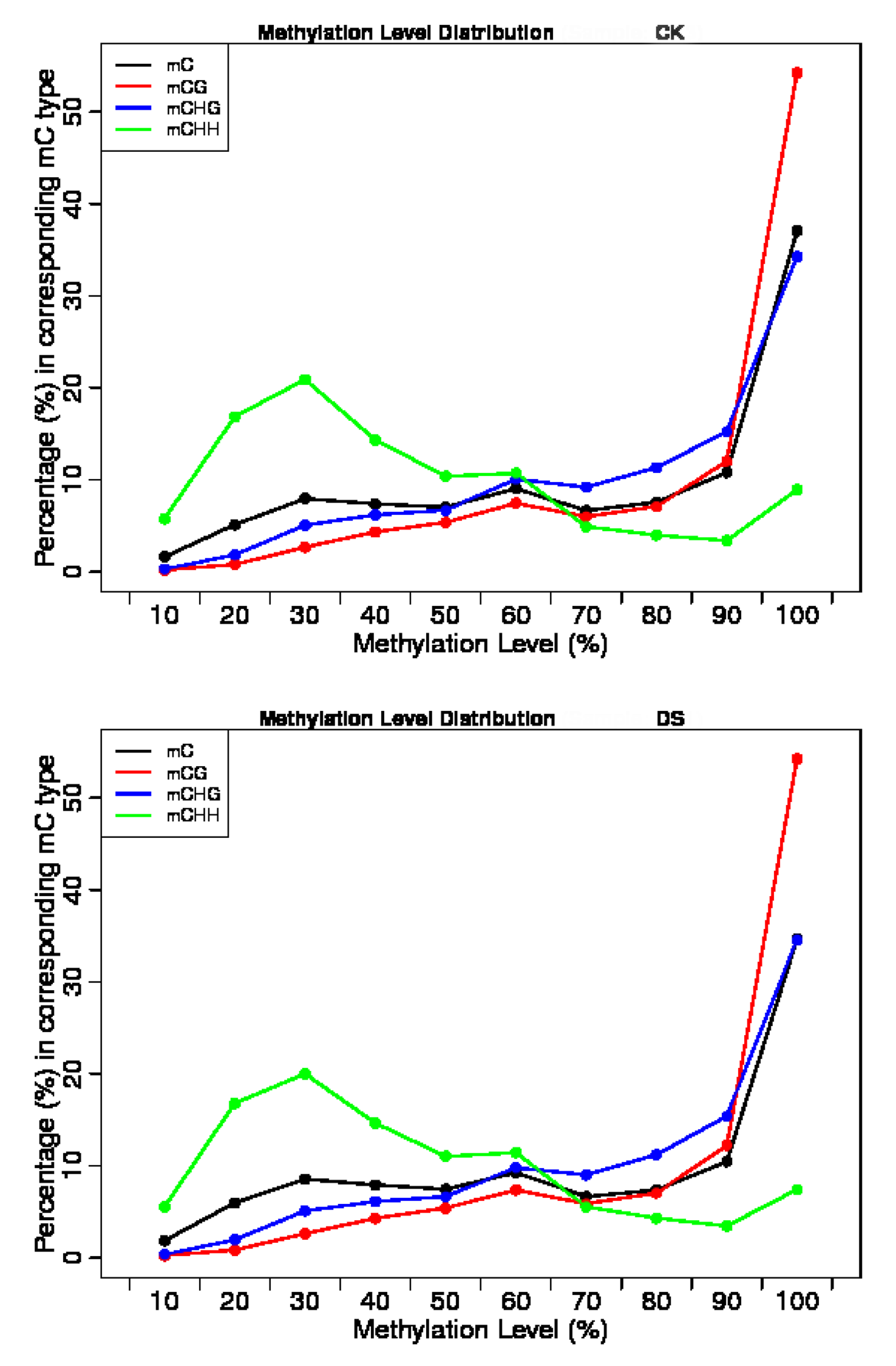

Supplement: Supplementary file 1 — Supplementary Information. [file 41598_2020_64975_MOESM1_ESM.zip › Supplementary information_Revised/FigureS6.tif]
